# Differential roles of *Cassia tora 1-deoxy-D-xylulose-5-phosphate synthase* and *1-deoxy-D-xylulose-5-phosphate reductoisomerase* in trade-off between plant growth and drought tolerance

**DOI:** 10.3389/fpls.2023.1270396

**Published:** 2023-10-20

**Authors:** Chunyao Tian, Huige Quan, Ruiping Jiang, Qiaomu Zheng, Sipei Huang, Guodong Tan, Chaoyue Yan, Jiayu Zhou, Hai Liao

**Affiliations:** School of Life Science and Engineering, Southwest Jiaotong University, Chengdu, Sichuan, China

**Keywords:** *Cassia tora*, Leguminosae, isoprenoids, drought, trade-off, 1-deoxy-D-xylulose-5-phosphate synthase, 1-deoxy-D-xylulose-5-phosphate reductoisomerase

## Abstract

Due to global climate change, drought is emerging as a major threat to plant growth and agricultural productivity. Abscisic acid (ABA) has been implicated in plant drought tolerance, however, its retarding effects on plant growth cannot be ignored. The reactions catalyzed by 1-deoxy-D-xylulose-5-phosphate synthase (DXS) and 1-deoxy-D-xylulose-5-phosphate reductoisomerase (DXR) proteins are critical steps within the isoprenoid biosynthesis in plants. Here, five *DXS* (*CtDXS1-5*) and two *DXR* (*CtDXR1-2*) genes were identified from *Cassia tora* genome. Based on multiple assays including the phylogeny, *cis-*acting element, expression pattern, and subcellular localization, *CtDXS1* and *CtDXR1* genes might be potential candidates controlling the isoprenoid biosynthesis. Intriguingly, *CtDXS1* transgenic plants resulted in drought tolerance but retardant growth, while *CtDXR1* transgenic plants exhibited both enhanced drought tolerance and increased growth. By comparison of *β*-carotene, chlorophyll, abscisic acid (ABA) and gibberellin 3 (GA_3_) contents in wild-type and transgenic plants, the absolute contents and (or) altered GA_3_/ABA levels were suggested to be responsible for the balance between drought tolerance and plant growth. The transcriptome of *CtDXR1* transgenic plants suggested that the transcript levels of key genes, such as *DXS*, *9-cis-epoxycarotenoid dioxygenases* (*NCED*), *ent-kaurene synthase* (*KS*) and etc, involved with chlorophyll, *β*-carotene, ABA and GA_3_ biosynthesis were induced and their contents increased accordingly. Collectively, the trade-off effect induced by *CtDXR1* was associated with redesigning architecture in phytohormone homeostasis and thus was highlighted for future breeding purposes.

## 1 Introduction

Drought stress is one of the primary factors limiting the global geographic distribution of plants as well as their growth and productivity (Fang and Xiong, 2015). To counteract drought stress, plants have evolved a range of response mechanisms, one of which is the phytohormone-mediated pathway. Abscisic acid (ABA) is the main hormone that plants use in response to drought stress and plays a key role in plant growth and development (Lee and Luan, 2012). ABA together with stress response transcription factors, including DREBs and AREBs, trigger various physiological processes and metabolic changes that allow whole tissues to acquire drought tolerance (Muhammad Aslam et al., 2022). However, over the years, there has been increasing lines of evidence of delayed effects of ABA on normal plant growth (De Smet et al., 2003; Sreenivasulu et al., 2012). In *Arabidopsis*, the reduction in ABA content promoted root growth (Miao et al., 2021). Conversely, exogenous application of ABA inhibited lateral root emergence (De Smet et al., 2003), rhizoidal infection, and nodule formation (Xu et al., 2021). Similarly, overexpression of ABA-induced MAPKs resulted in seedlings and leaf senescence (Matsuoka et al., 2018). Overexpression of protein phosphatase 2C (PP2C), a negative regulator of ABA signaling, increased plant biomass production mainly due to accelerated inflorescence stem growth (Sugimoto et al., 2014). Thus, a key challenge for the development of next-generation agriculture is to balance agro-industrial needs (enhanced plant growth yield) and plant adaptation during the growing season (tolerance to abiotic stresses), and ABA antagonistic regulators have been further introduced to address this challenge (Zhang et al., 2022).

The plant hormone gibberellin (GA) is generally considered a negative regulator of ABA and mediates many physiological processes antagonistically with ABA (Shu et al., 2018). ABA and GA are 15- and 20-carbon plant hormones, respectively, and can be broadly attributed to isoprenoids. In addition to ABA and GA, isoprenoids also include photosynthetic pigments (chlorophyll and carotenoid), electron transporters (plastoquinone and ubiquinone), structural components of membranes (phytosterols), and anti-pathogen agents (phytoalexins), which play a role in nutrient accumulation, photosynthesis, plant growth and development and adaptation to environmental stresses. In addition to these plant-specific functions, many plants isoprenoids have been shown to be of industrial and therapeutic interest. The plant-produced tetraterpene *β*-carotene (provitamin A) is one of basic nutrients needed to maintain human health. Intriguingly, the terpenoids and sesquiterpenes produced by rhizosphere-inhabiting bacteria/fungi have been reported to potentially promote plant growth and stress tolerance (Tyagi et al., 2018). In plants, isoprenoid is synthesized mainly by sequential condensation and modification of two common building-blocks, isopentenyl diphosphate (IDP) and dimethylallyl diphosphate (DMADP), via the mevalonic acid (MVA) pathway in the cytosol and the 2-methyl-d-erythritol-4-phosphate (MEP) pathway in the plastid. From the common intermediate, geranylgeranyl diphosphate (GGPP), the biosynthetic pathways of GA and ABA diverge (Rodríguez-Concepción and Boronat, 2015). Since GA and ABA share common upstream biosynthetic pathways and common intermediate, the genetic manipulation on the genes in the upstream biosynthetic pathways might simultaneously increase the GA and ABA contents. In the MEP pathway, 1-deoxy-D-xylulose-5-phosphate synthase (DXS) and 1-deoxy-D-xylulose-5-phosphate reductoisomerase (DXR) catalyze the first and second steps, respectively. In previous studies, DXS and DXR were considered as different regulators of fluxes during the formation of various isoprenoids (You et al., 2020) and their roles remained elusive. Specifically, both AtDXS and AtDXR have been suggested to be rate-limiting enzymes that increase carotenoid, chlorophyll, and ginkgolide content (Estévez et al., 2001; Carretero-Paulet et al., 2006; Kim et al., 2006), yet only DXS but not DXR caused increases in carotenoids and chlorophyll in *Daucus carota* or rice (Simpson et al., 2016; You et al., 2020), and increases in ABA and GA in poplars (Wei et al., 2019). In transgenic *Arabidopsis* plants with overexpressed *DXS*, the isoprenoid products were not markedly increased due to diversion of flux via 2-C-methylerythritol-2,4-cyclodiphosphate (MEcDP) (Wright et al., 2014). On the other hand, overexpression of the *Lilium DXR* gene has also been reported to increase monoterpene content in tobacco flowers (Zhang et al., 2018). In this way, plant *DXS* and *DXR* genes have been suggested to function in isoprenoid metabolism in a species- or organ-specific manner, highlighting the significance of elucidating the distinct roles of *DXS* and *DXR* genes in various plant species (You et al., 2020).

*Cassia tora* L. (Leguminosae), also known as *Senna tora*, is an important herb that grows mainly in Asia and Africa and has attracted attention for centuries due to its widespread use as a traditional medicine and food ingredient. Consumer awareness and knowledge of the health benefits of *C. tora* have greatly stimulated the marketing and consumption of *C. tora* products, which contain a variety of bioactive compounds such as betulinic acid, flavonoids, rotenoids, stigmasterol and *β*-carotene (Vats and Kamal, 2014; Vats, 2018; Górnaś et al., 2019). Besides, *C. tora* contains a large amount of anthraquinones whose part of backbone is biosynthesized via the MEP pathway (Liu et al., 2014). However, few studies on genes involved in MEP pathway in *C. tora* have been reported, mainly due to the lack of the *C. tora* genome. Recently, thorough genomic analysis and functional identification of candidate genes involved in the MEP pathway of *C. tora* became possible due to the availability of genomic data of *C. tora* (Kang et al., 2020). Therefore, the objectives of this study were (1) to perform an in-depth analysis of the *DXS* and *DXR* genes of *C. tora* (ASM1485142v1) by structural and functional annotation. Accordingly, the *CtDXS1* and *CtDXR1* genes, which may play a role in isoprenoid biosynthesis, were selected as candidates for further studies; (2) to investigate the different roles of the *CtDXS1* and *CtDXR1* genes in isoprenoid biosynthesis, plant growth, and drought tolerance; and (3) to put forward the potential molecular mechanism to achieve a trade-off between drought tolerance and plant growth. As a consequence, this report provides, for the first time, a basis for further investigation of the biological functions of *DXS* and *DXR* genes in *C. tora*, which can be used to develop genomic strategies for next-generation agriculture.

## 2 Results

### 2.1 Genome-wide identification of DXS and DXR genes in *C. tora*

DXSs and DXRs are key regulators of the MEP pathway and are conserved in the plant kingdom (Zeng and Dehesh, 2021). The genomes of *Arabidopsis*, soybean, and *Panicum virgatum* encode 3, 10, and 11 *DXS* genes, respectively, whereas the genome of algae contains only one *DXS* gene copy (de Luna-Valdez et al., 2021). In *Arabidopsis*, rice and *Coffea arabica*, DXR is encoded by a single-copy gene (Silva et al., 2020). In this study, a genome-wide analysis of the *C. tora* genome was performed to identify *DXS* and *DXR* genes. There are two main methods for identifying DXS and DXR family genes. In the first approach, a BlastP search was performed in the *C. tora* genome database using hidden Markov model (HMM) profiles of known DXS and DXR sequences. In the second method, NCBI’s conserved domain database (CDD) was performed to validate DXS and DXR members. After removing redundant sequences, a total of five *DXS* and two *DXR* genes were identified in *C. tora* (**Table 1**). Gene names, sequence numbers, protein sizes, molecular weights and chromosomal locations are listed in **Table 1**.

**Table 1:** List of the identified *DXS* and *DXR* family members in *C. tora*.

Gene Name	Sequence ID	Size (AA)/Mw (kDa)	Chromosomal localization
*CtDXS1*	KAF7813697.1	708/76.544	*CM026269.1*
*CtDXS2*	KAF7829270.1	700/75.489	*CM026265.1*
*CtDXS3*	KAF7838070.1	731/78.773	*CM026262.1*
*CtDXS4*	KAF7817227.1	720/78.967	*CM026268.1*
*CtDXS5*	KAF7837172.1	713/77.026	*CM026262.1*
*CtDXR1*	KAF7806089.1	471/51.290	*CM026271.1*
*CtDXR2*	KAF7825848.1	465/50.547	*CM026265.1*

**Figure 1:**
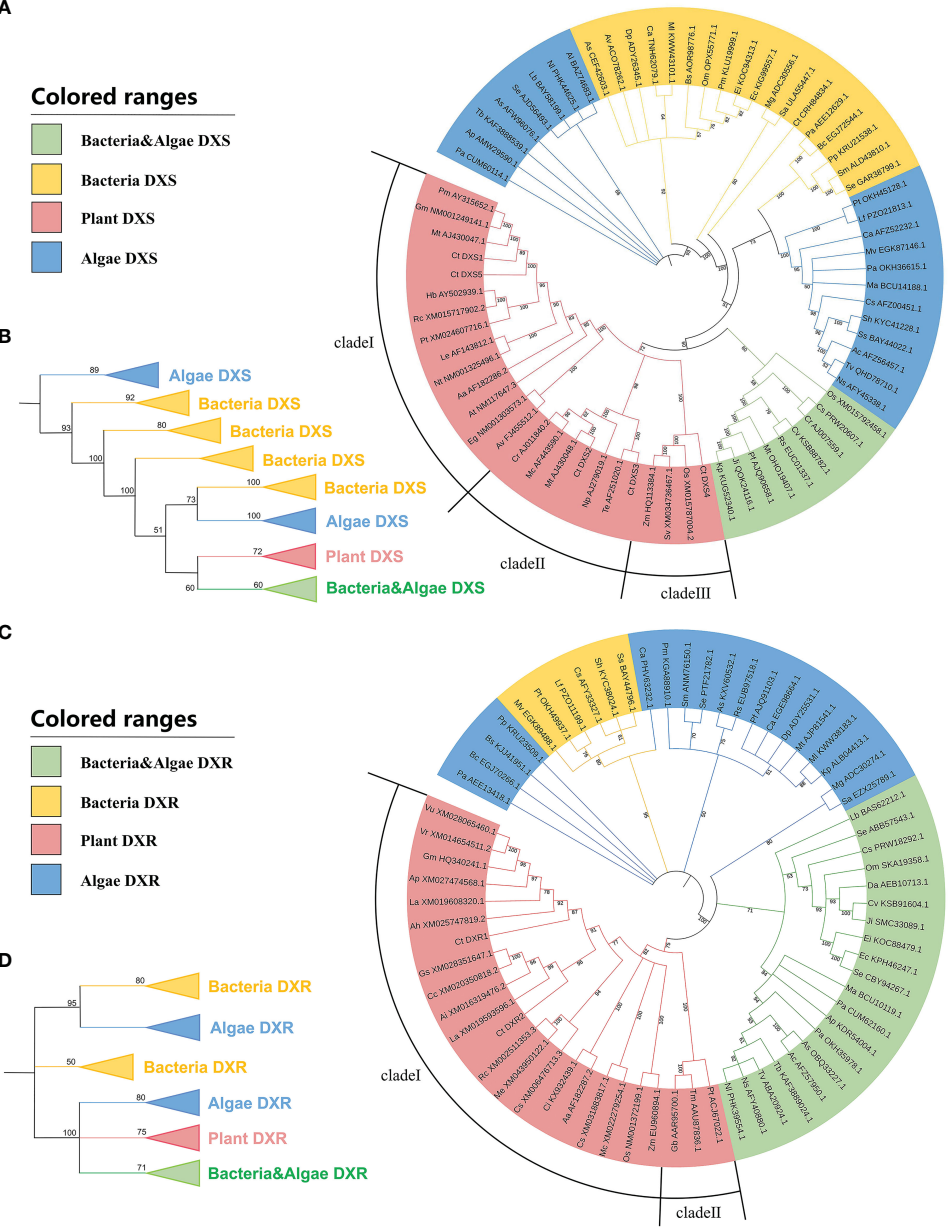
Phylogenetic analysis of the *DXSs* and *DXRs*. The bootstrap values were represented above the nodes. **(A)** NJ tree of *DXSs* from various organisms. **(B)** Topological structure of the NJ tree of *DXSs*. **(C)** NJ tree of *DXRs* from various organisms. **(D)** Topological structure of the NJ tree of *DXRs*.

The nucleotide sequences of five *CtDXS* genes were phylogenetically analyzed with the plant, algae, and bacteria *DXS* sequences. Consistent with previous reports (You et al., 2020; de Luna-Valdez et al., 2021), plant *DXS* was clustered into three major clades, namely, clade I including *CtDXS1* and *CtDXS5*, clade II including *CtDXS2* and *CtDXS3*, and clade III including *CtDXS4*. All *DXS* clades were monophyletic clusters with highly supported bootstrap (100, 86 and 100 BP, respectively). Furthermore, our phylogenetic results indicated that *DXSs* from the same species, such as *Oryza sativa* (abbreviated as Os in **Figure 1**), *Medicago truncatula* (abbreviated as Mt in **Figure 1**), and *C. tora*, were distributed in different clades, suggesting that the replication that gave rise to these different clades occurred prior to the divergence of these species. Members of clade I have been reported to play important roles in the biosynthesis of housekeeping and photosynthetic terpenoids such as chlorophylls and carotenoids, whose gene expression is dependent on light conditions (You et al., 2020). Members in clade II play a minor ecological role in the production of functional terpene metabolites, whereas clade III members are associated with postembryonic development in *Arabidopsis* and are not involved in primary metabolism and isoprenoid synthesis (de Luna-Valdez et al., 2021). In phylogenetic analysis (**Figure 1A**), *CtDXS1* was closely related to legumes and had the highest sequence similarity to *MtDXS1* (AJ430047.1), which encoded an enzyme with significant DXS activity, i.e., it catalyzed glyceraldehyde 3-phosphate and pyruvate to produce DXP (Walter et al., 2002), so it was subsequently selected to study its effect on isoprenoid biosynthesis. Based on the results of amino acid comparison and classification of conserved structural domains, the CtDXS1 protein belongs to the transketolase superfamily. For example, similar to other DXSs, CtDXS1 contains a TPP-binding domain (coenzyme of DXS) in motif 10 at the N-terminal end with the highly conserved sequences -GDG- and -LNDN- at the starting and end points, respectively (**Figure S1**). In addition, the His residue at position 93 (H93) in motif 5 at the N terminus of CtDXS1 showed a good match with His49 in *Escherichia coli* DXS, which has been reported to be involved in the catalytic action of DXS (Yang et al., 2012c). Invariant Asp294 residue in motif 7 was also found in the intermediate region of CtDXS1, and is thought to be specific for the enzymatic activity (Crowell et al., 2003). In addition, a DRAG structural domain, which is thought to play an important role, was found in motif 2 at its C-terminus, which starts and ends with the sequences -HCGS- and -PSD, respectively, similar to other plant DXSs. The highly conserved H499 was proposed to interact with glyceraldehyde 3-phosphate (Carretero-Paulet et al., 2013).

*C. tora DXRs* included two members (KAF7806089.1 and KAF7825848.1) that were phylogenetically assigned to plant *DXR* clade I, which included the legumes *Glycine max* (abbreviated as Gm in **Figure 1**) and *M. truncatula* (**Figure 1C**). In respect to *CtDXR2*, *CtDXR1* was more closely related to *GmDXR1* (accession no HQ340241.1), which played a role in biosynthesis of isoprenoids, i.e. *GmDXR1*-overexpressing plants exhibited increasing isoprenoid content (Zhang et al., 2012). The enzyme consists of three distinct structural domains: an N-terminal NADPH-binding domain, a central linker domain and a C-terminal helical structural domain (Proteau, 2004). Since NADPH is involved in the catalytic reaction of DXR, the NADPH-binding structural domain is essential for the catalytic activity of DXR. Two highly conserved NADPH binding regions, GSTGS(I/V)GT and LAAGSN(V/I), are identified in motif 5 in the N-terminal structural domain of CtDXR1, while two substrate binding regions, LPADSEHSAI in motif 4 and NKGLEVIEAHY in motif 2, located in its central linker domain, are highly homologous to DXRs from other plants (**Figure S2**).

Furthermore, as an annual land plant, *C. tora* shows a symbiotic relationship with bacterial endophytes (Kumar et al., 2015). To confirm whether the *CtDXS1* and *CtDXR1* genes were indeed derived from *C. tora*, we performed a series of exercises. The first thing we did was to search for homologous regions of both genes using Blastn. The highest scoring sequences were *DXS* from *Prosopis alba* (XP_028781649.1), and *DXR* from *Prosopis alba* (XM_028922493.1), respectively. In addition, phylogenetic analysis using nucleotide sequences of *DXSs* and *DXRs* from plants and microorganisms demonstrated that *CtDXS1* and *CtDXR1* were clustered into plant clade (**Figures 1B, D**). Third, the average identity of *CtDXS1* with plant *DXSs* was approximately 5% higher than that with microorganism *DXSs*. while the average identity of *CtDXR1* with plant *DXRs* was approximately 9% higher than that with microorganism *DXRs*, respectively. The sequences of *CtDXS1* and *CtDXR1* were much more similar to those of other plants, while both were far from those of microorganisms. Therefore, it was inferred that *CtDXS1* and *CtDXR1* were genuine member from *C. tora* and not endophytes.

### 2.2 Cis-acting elements analysis of CtDXS and CtDXR genes

**Figure 2:**
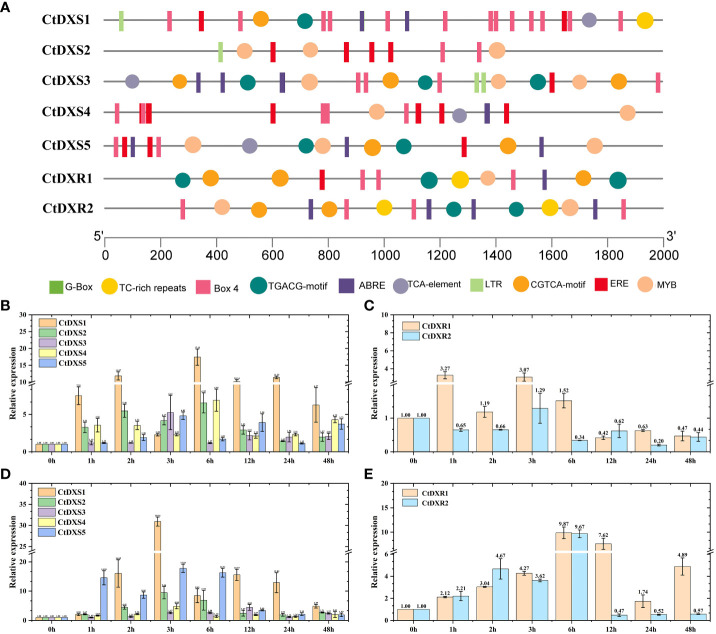
Cis-element analysis and expressional pattern of *CtDXS* and *CtDXR* genes. **(A)** Cis-acting elements of *CtDXS* and *CtDXR* genes. Real-time PCR of *CtDXS* **(B)** and *CtDXR* **(C)** genes under simulated drought treatment. Real-time PCR of *CtDXS* **(D)** and *CtDXR* **(E)** genes under ABA treatment. To normalize the relative expression level, *Elongation factor 1a* was used as an internal control.

The identification of *cis*-acting elements may help to determine the molecular functions of *CtDXS* and *CtDXR* genes. Based on the corresponding gene sequences, *cis*-acting elements were obtained using PlantCare (http://bioinformatics.psb.ugent.be/webtools/plantcare/html/) analysis. The results showed that the promoters of the *CtDXS1*, *CtDXS3*, *CtDXS5*, *CtDXR1* and *CtDXR2* genes have multiple *cis*-acting elements involved in stress responses and hormones (**Figure 2A**). These include ABRE (response to ABA, ACGTG), MYB (response to stress, GA and ABA, TAA CCA), CGTCA-motif (response to stress and MeJA, CGTCA), and TGACG-motif (response to stress and MeJA, TGACG). In contrast, the promoter of the *CtDXS2* gene lacks the ABRE and TGACG-motif, and the promoter of the *CtDXS4* gene lacks the TGACG-motif. Therefore, we can infer that these five genes with multiple response elements may be involved in plant responses to abiotic stresses.

### 2.3 The expressional pattern of CtDXS and CtDXR genes

As shown in **Figures 2B–E**, both *CtDXS1* and *CtDXR1* genes were sensitive under drought and ABA treatments. Under drought treatment, the expression level of *CtDXS1* gene gradually increased and reached the highest expression level (17.42-fold) at 6 h (**Figure 2B**), whereas the expression level of *CtDXR1* gene increased rapidly and reached the highest expression level at 1 h (3.27-fold), compared with the control without treatment, respectively (**Figure 2C**). Under ABA treatment, the *CtDXS1* gene showed a similar trend to the drought treatment, reaching its highest expression level at 3 h (30.92-fold) (**Figure 2D**), while the highest expression level of the *CtDXR1* (9.87-fold) gene occurred at 6 h (**Figure 2E**). The different expression patterns of the *CtDXS1* and *CtDXR1* genes imply that these two genes may play different roles in the drought and ABA responses. Compared with the *CtDXS1* gene, the other four *CtDXS* genes were expressed at relatively lower levels under drought and ABA treatments, respectively. The *CtDXR2* gene was also expressed at lower levels than *CtDXR1* under drought and ABA treatment conditions, respectively. Therefore, based on the phylogenetic tree, *cis*-acting element analysis, and expression patterns under various stresses, the *CtDXS1* and *CtDXR1* genes are promising for drought response and were therefore selected for further functional identification.

### 2.4 Subcellular localization of CtDXS1 and CtDXR1 proteins

TARGETP showed a putative chloroplast transfer peptide in CtDXS1 with a high probability of 0.8976, whereas ChloroP predicted that CtDXS1 is not located in chloroplasts. Considering the subcellular localization of the MEP pathway in plants (Llamas et al., 2017; Zhang et al., 2019), it is a better prediction that CtDXS1 is located in chloroplasts. The predicted chloroplast transfer peptide at sites 46-47 of CtDXS1 has a conserved VX-A pattern cleavage site (for CtDXS1, X: C, **Figure S1**). Meanwhile, there was a putative chloroplast transit peptide at sites 48-49 (**Figure S2**) of CtDXR1 with a conserved CS-X patterned cleavage site (for CtDXR1, X: V), suggesting that CtDXR1 is preferentially located in chloroplasts.

**Figure 3:**
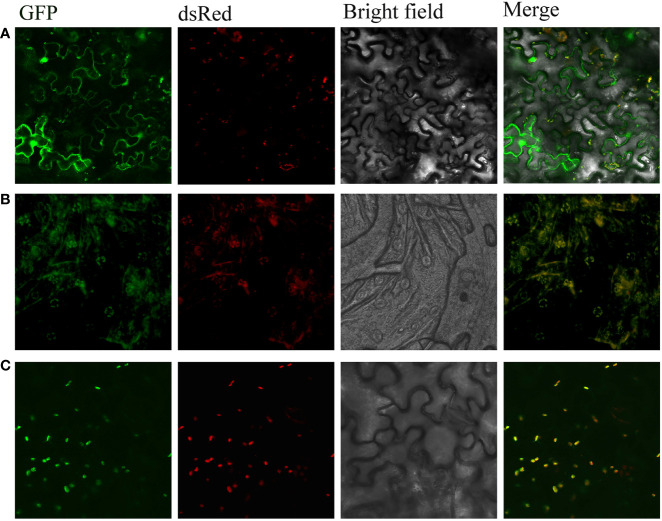
CtDXS1 and CtDXR1 were located in chloroplast. **(A)** Expression of *35S*: *GFP*, **(B)** Expression of *35S*: *CtDXS1*-*GFP*, **(C)** Expression of *35S*: *CtDXR1*-*GFP* in *N. benthamiana* is localized in yellow dots around the chloroplast, indicating the subcellular location of the fusion proteins. Free DsRed marks the chloroplast. Free GFP serves as a localization control.

To verify the predicted subcellular location of CtDXS1 and CtDXR1 proteins, pBI121 *CtDXS1: GFP* and pBI121 *CtDXR1: GFP* expression vectors were transiently transformed into *N. benthamiana* leaves, respectively. Under laser confocal scanning, chloroplasts and GFP showed red and green fluorescence, respectively. The subcellular localization of CtDXS1 and CtDXR1 proteins was able to be observed based on the combined fluorescence, where the visualized yellow fluorescence was evident, indicating that both CtDXS1 and CtDXR1 are located in chloroplasts (**Figure 3**), in which the MEP pathway is located.

### 2.5 Production of transgenic plants

DXS and DXR catalyze the first and second steps of the MEP pathway and are considered to be the rate-limiting enzymes for isoprenoid production in bacteria and plants (Estévez et al., 2001; Liao et al., 2023). To verify the role of these two genes in influencing the level of plastid isoprenoids in plants, recombinant plasmids containing *CtDXS1* and *CtDXR1* ORFs, respectively, controlled at their 5` ends by the CaMV *35S* promoter, were constructed. Both recombinant plasmids were introduced into *N. benthamiana* by *Agrobacterium*-mediated transformation, respectively. Transgenic plants were selected on media containing kanamycin. Transgenic plants exhibited a green phenotype similar to that of wild-type plants. The T2 lines were analyzed for gene expression, isoprenoid content and phenotype.

To determine the expression of *CtDXS1* and *CtDXR1*, reverse-transcription PCR (RT-PCR) analysis was performed on 20-day-old seedlings of transgenic and wild-type plants using cDNA as template. As shown in **Figure S3**, *CtDXS1* and *CtDXR1* were substantially expressed in *CtDXS1* transgenic plants and *CtDXR1* transgenic plants, respectively, while *CtDXS1* and *CtDXR1* were not detected in wild-type plants.

### 2.6 CtDXS1 and CtDXR1 transgenic plants increased isoprenoid contents

To determine the amounts of isoprenoids, four kinds of plastidic isoprenoids, such as chlorophyll, *β*-carotene, ABA and GA_3_ were selected. The *β*-carotene in *CtDXS1* transgenic plants was 1.10 ± 0.10-fold higher than that in wild-type plants, while plants overexpressing *CtDXR1* gene presented a 2.29 ± 0.12-fold increase of *β*-carotene relative to that in the wild-type plants (*p*<0.01) (**Figure S4A**). As shown in **Figure S4B**, total chlorophyll content in *CtDXS1* transgenic plants achieved a 1.34 ± 0.03-fold increase, compared to the wild-type levels (*p*<0.01). In *CtDXR1* transgenic plants, a total of chlorophyll content was observed to be a 1.46 ± 0.03-fold increase in respect to that in wild-type plants (*p*<0.01). It would be of particular interest to explore the effect of altering the expression of *CtDXS1* and *CtDXR1* on GA levels. Since it has been shown that GA_3_ plays a central role in plant development (Jaudal et al., 2018), its level was used as a marker of GA levels in this study. As shown in **Figure S4C**, for those *CtDXR1* transgenic plants, the levels of GA_3_ were detected to be 3.53 ± 0.09-fold higher than the wild-type levels. In *CtDXS1* transgenic plants, lower levels of GA_3_ were observed to be 6.56 ± 0.2% of the wild-type levels. Since a major part of ABA biosynthesis is carried out in the plastid from *β*-carotene, overexpression of *CtDXS1* and *CtDXR1* may affect the levels of this phytohormone. It was observed that *CtDXS1*-overexpressing plants accumulated a 4.32 ± 1.19-fold increase of ABA content compared to wild-type levels (*p*<0.01). In *CtDXR1*-overexpressing plants, the ABA content was 3.77 ± 0.84-fold higher than the wild-type levels (*p*<0.01) (**Figure S4D**).

### 2.7 GA_3_ and ABA played role in plant growth and drought tolerance

In order to determine whether two isoprenoid-type phytohormones, GA_3_ and ABA, played role in plant growth and drought tolerance, the GA_3_ and ABA were exogenously applied to wild-type plants, respectively. The phenotypes were recorded after treatment of 7 days. As shown in **Figure S5** and **Table S1**, exogenous GA_3_ application improved plant growth since the plants treated by GA_3_ exhibited larger leaf size (1.51 cm^2^, *p*<0.05), number of lateral roots (14.2, *p*<0.05), fresh weight (111.4 mg, *p*<0.05) and length of taproots (96 mm) in respect to control plants with (0.90 cm^2^, 9.8, 86.08 mg and 90.6 mm), respectively. These key results support the critical importance of GA_3_ in explaining the growth phenotype, but also imply the importance of phytohormone ratio for plant growth.

Meanwhile, exogenous ABA application enhanced drought tolerance of wild-type plants. After drought treatment for 7 days, the plants treated by ABA had a higher value of height (71 mm; *p*<0.05), number of lateral roots (9.4), length of taproots (58.6 mm, *p*<0.05), fresh weight (36.26 mg, *p*<0.05), but similar number of leaves (7) compared to control plants with (56.8 mm, 8.2, 44.4 mm, 26.98 mg and 6.8), respectively (**Figure S5**, **Table S2**).

### 2.8 CtDXS1 and CtDXR1 transgenic plants increased defense-related enzymes activities

To elucidate the plant tolerance induced by ABA accumulation, further biochemical analyses were performed. The activities of phenylalanine ammonia lyase (PAL), peroxidase (POD) and superoxide dismutase (SOD) were measured using 30-day-old leaves of wild-type and transgenic plants. As a result, transgenic plants showed enhanced activities of PAL, POD and SOD.

As shown in **Figure S6**, total PAL activity was increased in *CtDXS1* transgenic plants, up to 1.51 ± 0.03-fold higher than wild-type levels. In *CtDXR1* transgenic plants, a total PAL activity was observed to be a 3.12 ± 0.10-fold increase relative to wild-type levels (*p*<0.05).

Overexpression of *CtDXS1* and *CtDXR1* genes also resulted in increased POD activity. Relative to wild-type levels, *CtDXS1* transgenic plants showed a 2.23 ± 0.31-fold increase of total POD activity. Plants overexpressing *CtDXR1* contained a 4.66 ± 0.54-fold higher total POD activity than wild-type levels (*p*<0.05).

As shown in **Figure S6**, *CtDXS1* transgenic plants had SOD levels of a 1.25 ± 0.02-fold increase compared to wild-type levels (*p*<0.01). For those transgenic plants that accumulated *CtDXR1*, SOD activity was 1.59 ± 0.03-fold higher than that in wild-type plants (*p*<0.001).

### 2.9 Phenotypes of transgenic plants

**Figure 4:**
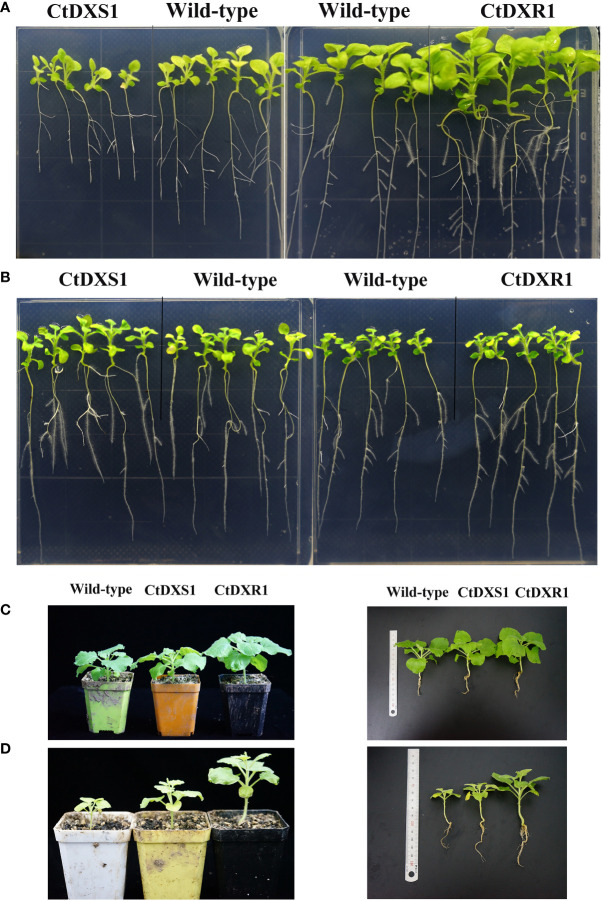
Phenotypes of the *CtDXS1* transgenic, *CtDXR1* transgenic and wild-type plants. **(A)** The 20-day-old seedlings of *CtDXS1* transgenic, *CtDXR1* transgenic and wild-type plants cultivated on culture medium under normal conditions; **(B)** The 20-day-old seedlings of *CtDXS1*-transgenic, *CtDXR1*-transgenic and wild-type plants cultivated on culture medium under drought stress; **(C)** The 60-day-old seedlings of *CtDXS1* transgenic, *CtDXR1* transgenic and wild-type plants cultivated in soil mixture under normal conditions; **(D)** The 60-day-old seedlings of *CtDXS1* transgenic, *CtDXR1* transgenic and wild-type plants cultivated in soil mixture under drought stress.

To determine whether the observed changes in isoprenoid levels affect plant morphology, the plants of wild-type, *CtDXS1*, and *CtDXR1* transgenic plants were grown and their phenotypes were compared under normal conditions. No significant differences in general plant morphology were observed between our transgenic plants and the wild-type control, except for biomass. To explore this phenotype more closely, the biomass of the transgenic plants was estimated at the seedling stage (20 days) by leaf size, number and length of lateral roots. It was observed (**Figure 4A**, **Table 2**) that the weight of wild-type plants had 100.02 mg, while those of *CtDXS1* and *CtDXR1* transgenic plants were 83.95 mg (*p*<0.05) and 112.48 mg (*p*<0.05), respectively (**Figure 4A**, **Table 2**). The *CtDXR1* transgenic plants had a greater number of lateral roots (19.6, *p*<0.05), while wild-type and *CtDXS1* transgenic plants had fewer number of 15.1 and 10.4, respectively. In addition, the highest length of total lateral roots was observed of 77.20 mm (*p*<0.001) in *CtDXR1* transgenic plants, while those in wild-type and *CtDXS1* transgenic plants were relatively lower of 57.70 and 44.80 mm (*p*<0.05), respectively.

**Table 2:** Phenotypic statistics of wild-type and transgenic plants under normal conditions.

Plants	Weight (mg)	Number of leaves	Number of lateral roots	Length of lateral roots (mm)
Wild-type	100.02±1.73	7.70±0.14	15.1±0.87	57.70±0.20
*CtDXS1*	83.95±4.65*	7.40±0.22*	10.4±1.28	44.80±0.33*
*CtDXR1*	112.48±3.34*	7.60±0.22	19.6±0.73*	77.20±0.31***

* and *** represented statistical significance of p<0.05 and p<0.001, respectively.

Intriguingly, both *CtDXS1* and *CtDXR1* transgenic plants exhibited higher drought tolerance than wild-type plants at seedling stage. After drought stress induced by 200 mM mannitol for 10 days, the leaves of the transgenic plants remained green and unfold, while those of some wild-type seedlings curled up, implying that the wild-type plants may be in a dehydrated state (**Figure 4B**). Compared with wild-type plants, the *CtDXS1* and *CtDXR1* transgenic plants showed greater height and fresh weight, and higher number of lateral root and leaf, respectively (**Table 3**). Therefore, in our definition both *CtDXS1* and *CtDXR1* transgenic plants may be able to improve tolerance to drought stress.

**Table 3:** Phenotypic statistics of seedlings cultivated on culture medium under drought stress.

Plants	Height (cm)	Number of leaves	Number of lateral roots	Weight (mg)
Wild-type	1.73±0.16	7.00±0.2	6.2±0.77	26.18±2.18
*CtDXS1*	2±0.23	8.00±0***	11.2±0.77**	51.96±4.91**
*CtDXR1*	2.16±0.15*	8.20±0.18*	10.60±0.54*	60.48±2.97***

*, ** and *** represented statistical significance of p<0.05, p<0.01 and p<0.001, respectively.

Meanwhile, to investigate the drought tolerance of transgenic and wild-type plants at their mature stages, 30-day-old seedlings were cultivated in pots containing a soil mixture (nutrient soil:vermiculite:peat, 1.5:1:1, V/V/V) and grown at 25°C, 16 h of light and 8 h of darkness. After 30 days, the plants under normal and drought conditions, respectively, were used to estimate the height of plants, the number of leaves, the number and length of lateral roots, average lateral and longitudinal lengths of leaves. We observed that the phenotypes of the potted plants showed similar tendencies to those of the plants on the culture medium. Under normal conditions, *CtDXR1* transgenic plants showed the highest height (12.8 cm, *p*<0.001) and leaf area (1453.38 mm^2^), while *CtDXS1* transgenic and wild-type exhibited similar height and leaf area (**Figure 4C**, **Table 4**). Under drought stress, the transgenic plants essentially exhibited greater drought tolerance than the wild-type plants. The *CtDXR1* transgenic plants exhibited the highest height (12.4 cm, *p*<0.01), lateral root length (59.5 mm, *p*<0.001), number of leaves (10, *p*<0.01) and largest leaf areas (789.13 mm^2^, *p*<0.001), respectively, while *CtDXS1* transgenic plants had higher height (11.4 cm, *p*<0.05), lateral root length (53.75 mm, *p*<0.001), number of leaves (8.75) and larger leaf areas (488.75 mm^2^) (**Figure 4D**, **Table 4**). Thus, our results suggested that transgenic plants were able to simultaneously improve drought tolerance in young and more mature plants.

**Table 4:** Phenotypic statistics of seedlings cultivated in soil mixture under normal conditions (NC) and drought stress (DS), respectively.

Plants	Height (cm)	Number of leaves	Length of lateral root (mm)	Average lateral length of leaves (mm)	Average longitudinal lengths of leaves (mm)
Wild-type (NC)	10.5±0.18	11.5±0.25	45.75±1.63	36.5±1.09	36.5±1.48
*CtDXS1* (NC)	11.9±0.31*	10.5±0.43	67±3.5**	37.75±1.24	37.75±0.96
*CtDXR1* (NC)	12.8±0.30***	10.5±0.43	65±2.1	38.5±1.15	37.75±0.96
Wild-type (DS)	7.0±0.20	7.75±0.41	30±0.35	12.75±0.89	13.75±1.14
*CtDXS1* (DS)	11.4±1.22*	8.75±0.22	53.75±5.72***	21.25±1.92	23±1.54
*CtDXR1* (DS)	12.4±0.25***	10±0.35**	59.5±2.17***	26.75±2.07	29.5±0.75***

*, ** and *** represented statistical significance of p<0.05, p<0.01 and p<0.001, respectively.

### 2.10 Transcriptomic alteration in CtDXR1 transgenic plants

**Figure 5:**
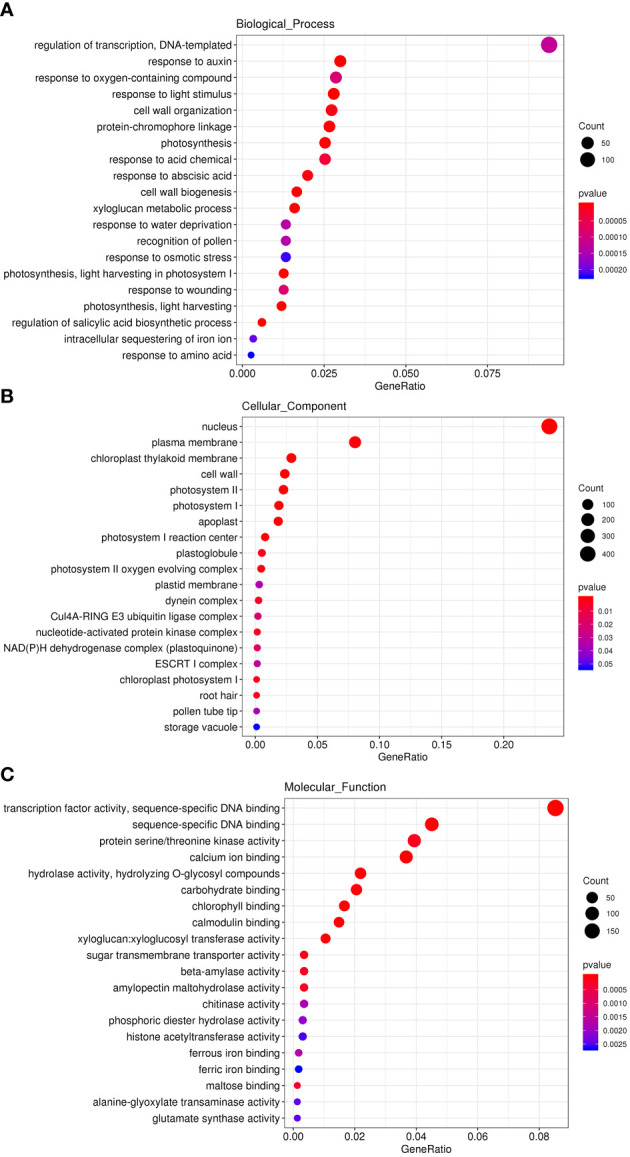
GO analyses of up-regulated genes in top 20 GO terms of Biological Process **(A)**, Cellular Component **(B)**, and Molecular Function **(C)**, respectively.

Since *CtDXR1* transgenic plants strike a balance between plant growth and drought tolerance, RNA-seq analysis was performed to reveal transcriptional changes in *CtDXR1* transgenic plants. Overall, wild-type and *CtDXR1* transgenic plants produced 44.63 (6.67 Gb) and 41.45 (6.21 Gb) clean reads, respectively. In total, 96.35% of clean reads with Q30 rate of at least 94.15% were mapped to the *N. benthamiana* genome, indicating that the transcriptome data was reliable. A total of 4,333 genes were up-regulated and 3,340 genes were down-regulated in the DEGs, with fold changes greater than 2 and Q-values less than 0.05. Gene ontology (GO) was further investigated and the top 20 GO terms of biological processes (BP), cellular components (CC) and molecular functions (MF) in the up-regulated genes were analyzed (**Figure 5**). Among the up-regulated genes, “response to abscisic acid”, “response to water deprivation” and “regulation of salicylic acid biosynthetic process” in biological process, as well as “calcium ion binding” and “calmodulin binding” in molecular functions indicated that the enhanced drought tolerance of *CtDXR1* transgenic plants might be involved with ABA-related pathway. The “xyloglucan metabolic process”, “cell wall biogenesis”, “cell wall organization” and “response to auxin” in biological process, “apoplast” in cellular component, as well as “carbohydrate binding” and “xyloglucan: xyloglucosyl transferase activity” in molecular functions were also consistent with the enhanced growth of *CtDXR1* transgenic plants. Photosynthesis is the most affected pathway of biological processes, cellular components, and molecular functions among the regulated genes, which is consistent with the increase in chlorophyll content.

### 2.11 Transcriptional alterations of genes responsible for isoprenoid biosynthesis

**Figure 6:**
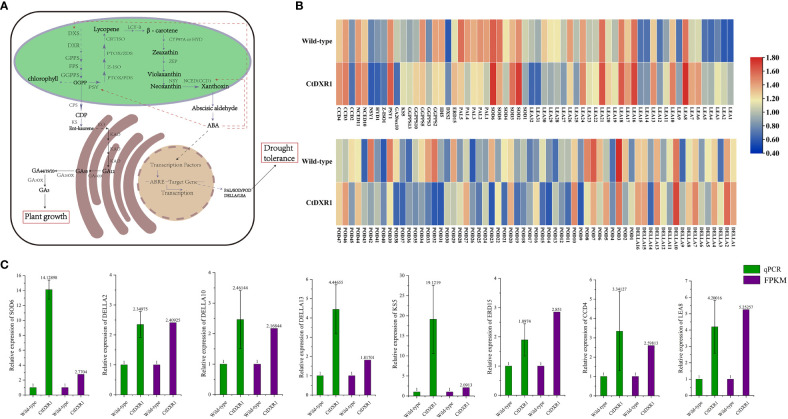
Isoprenoids biosynthesis and downstream signal transduction pathway. **(A)** Isoprenoids biosynthesis pathway is represented (adapted from Diretto et al., 2020 and Liu et al., 2015), with the differential expressed genes highlighted in heatmaps **(B)**. The real-time PCR results of partial differential expressed genes were represented in **(C)**. Error bars represent the standard error of the mean.

Previously, up-regulation of several genes involved in carotenoid biosynthesis was observed in *LCYB1*-overexpressing tobacco lines (Moreno et al., 2020). Therefore, the effect of *CtDXR1* on the expression of genes related to isoprenoid metabolism was analyzed in detail using *N. benthamiana*, which is closely related to tobacco (**Figure 6A**, **Table S3**). In total, 10 genes had increased transcript levels, including one *DXS*(*DXS2*), three *geranylgeranyl diphosphate synthases* (*GGPPS3, GGPPS10*, and *GGPPS13*), two *9-cis-epoxycarotenoid dioxygenases* (*NCED10* and *NCED11*), one *ent-kaurene synthase* (*KS5*), one *phytoene synthase* (*PSY1*), and two *carotenoid cleavage dioxygenases* (*CCD3* and *CCD4*). And the expression levels of eight genes were down-regulated, including two *GGPPSs* (*GGPP2* and *GGPP5*), one *isopentenyl diphosphate isomerase* (*IDI5*), one *gibberellin 20-oxidase* (*GA20ox10*), one *15-cis-zeta-carotene isomerase* (*Z-ISO1*), one *beta-carotene hydroxylase* (*HYD1*), one *neoxanthin synthase* (*NSY1*) and one *CCD* (*CCD2*) (**Figure 6B**, **Table S3**).

In the present study, many downstream genes regulated by ABA were significantly enhanced (**Figure 6B**, **Table S4**), such as three *DELLA proteins* (*DELLA2*, *10* and *13*), one *Late embryogenesis abundant protein* (*LEA8*), one *SOD* (*SOD6*), and an *early response to dehydration* (*ERD15*), whose expression was also confirmed by real-time PCR (**Figure 6C**, the nucleotide sequence and primers of each gene are listed in **Tables S5**, **S7**, respectively). In addition, *cis*-acting element analysis on six potential target genes in the *N. benthamiana* genome was performed using the sequence of the 2-kb segment upstream of ATG. It was found that the promoters of all six genes contain ABRE and (or) MYB, which are involved in ABA-dependent pathways under drought and salt stress (**Figure S7**, **Table S6**) (Yang et al., 2011). In addition, the G-boxes in the *DELLA2*, *DELLA13*, *ERD15* and *LEA8* promoters are also associated with the ABA-dependent pathway (Yang et al., 2012a). Therefore, the increased transcript levels of *SOD* were consistent with increased SOD activity in *CtDXR1* transgenic plants.

## 3 Discussion

### 3.1 Identification of DXS and DXR members in the C. tora genome

Since isoprenoids play a key protective role and are nutritionally important, the regulation of their production has been a target for agricultural improvement. In this study, four different isoprenoids were derived from the intermediate molecule GGPP, which is produced by condensation of IDP and DMADP units. DXS and/or DXR enzymes have all been shown to promote increased MEP-derived products when overproduced in different crop systems, including rice (You et al., 2020), carrot (Simpson et al., 2016) and barley (Walter et al., 2000). However, the relationship between drought tolerance and changes in plant isoprenoid-type hormones is still less clear. This is fundamentally different from plants transformed with genes encoding the CtDXS1 and CtDXR1 enzymes. In this study, a comprehensive genome-wide analysis of the *DXS* and *DXR* genes of *C. tora* was first done, and five *DXS* and two *DXR* genes were identified. The expansion of the *DXS* family was not limited to specific lineages, as these examples included both dicotyledonous and monocotyledonous plant species. In some cases, this expansion of the gene family was associated with genome-wide replication events that occurred in some of these species (like *Glycine max*) (Wang et al., 2021). In contrast, this is consistent with previous report (Silva et al., 2020) that *DXR* is a low-copy gene, with no more than two *DXR* genes in more than 96% of land plants, with the exception of *G. max* (three copies) and *Kalanchoe laxiflora* (four copies).

All published phylogenetic analyses of the DXS family agree on the existence of these three distinct groups, but they differ in the number of genes in the plant lineage. For example, the DXS2 group disappears in all crucifers, DXS3 in some Euphorbiaceae, and DXS1 is apparently absent in *Triticum aestivum* and *Arabidopsis halleri* (de Luna-Valdez et al., 2021). Consistent with previous report (You et al., 2020), our phylogeny shows that *DXS* genes from land plants clustered into three monophyletic clades (**Figure 1**) with high bootstrap support. Our phylogeny showed that these three clades included members from the same species (*Medicago truncatula* and *Oryza sativa*) (**Figure 1**), suggesting that the replication that gave rise to the different clades occurred prior to the divergence of these groups. More importantly, *CtDXS1* and *CtDXS5* were included in clade I involved in essential isoprenoid biosynthesis. The phylogenetic tree of *DXR* showed a more complex division than *DXS* (**Figure 1**). In the NJ tree, angiosperms were observed to be clustered in clade I, where legumes also appeared to be a strongly supported monophyletic group (91 BP). In clade II, gymnosperms were monophyletic and sister to angiosperms, similar to previous result (Tong et al., 2015). Since the two *CtDXR* genes were located in a monophyletic group and were sister to each other, it appeared that the duplication of the two *CtDXR* genes occurred after the divergence of *C. tora*.

Conservative *cis*-acting element assays indicated that the promoters of both *CtDXS1* and *CtDXR1* genes contained drought-responsive and ABA-responsive elements, suggesting that both genes might play a role in drought tolerance. This hypothesis was initially verified by real-time PCR, and the mRNA levels of both genes were increased under simulated drought and ABA treatments (**Figures 2B-E**). In general, genes with altered expression levels under environmental stress are thought to play a role in the stress response. Considering the phylogeny, *cis*-acting elements, expression pattern, and subcellular location detection, it was very likely that *CtDXS1* or *CtDXR1* genes influenced the biosynthesis of isoprenoid in plants.

### 3.2 The isoprenoid contents were responsible for the phenotypes of transgenic plants

In the next steps, four important plastidic isoprenoids, such as *β*-carotene, chlorophyll, GA3 and ABA, were analyzed. These isoprenoids derive via three pathways from the common GGPP intermediate, which is formed by condensation of IDP and DMADP units. *β*-carotene and ABA are downstream products of phytoene, a C40 intermediate molecule, via GGPP (Barja and Rodríguez-Concepción, 2021). Chlorophyll is a downstream product of phytyl pyrophosphate (phytyl-pp) via GGPP (Barja and Rodríguez-Concepción, 2021). GA constitutes a large family of diterpenoids that act as plant hormones and are involved in many developmental processes. These compounds are formed by the conversion of GGPP to *ent*-kaurene, and these initial steps are catalyzed by *ent*-copalyl diphosphate synthase (CPS) and *ent*-kaurene synthase (KS) (Su et al., 2016).

*β-*carotene, ABA and chlorophyll contents increased at different levels in *CtDXS1* and *CtDXR1* transgenic plants, except that *CtDXR1* transgenic plants had the highest GA_3_, while *CtDXS1* transgenic plants had the lowest GA_3_ content. It was particularly interesting that the relative change in ABA was greater than the observed change in total *β*-carotene. As shown in **Figures S4A, D**, the relative increase in *β*-carotene was more limited than the relative increase in ABA. Similar result was reported by Estévez et al. (2001) who found changes in ABA contents were greater than those observed for total carotenoids in *DXS*-overexpressing *Arabidopsis*. The reason for this may be attributed in part to the highly regulated and complex carotenoid biosynthetic pathway (Banerjee and Sharkey, 2014). For example, pro-vitamin A, which plays a central role in human growth, reproduction, immunity, and vision, is also derived from *β*-carotene (Spiegler et al., 2012). It is known that vitamin A and ABA are isolated from a common *β*-carotene precursor, so an increase in a particular product may not be reflected in the total isoprenoid levels of the transgenic plant. Also, ABA can be hydrolyzed back from its glucose-conjugated form to the active form by *β*-glucosidase (Xu et al., 2012). Furthermore, for a specific product, there are additional restrictive reaction steps, for example, 9-*cis*-epoxycarotenoid dioxygenase (NCED) catalyzes the formation of xanthoxin from neoxanthin and plays a key role in ABA biosynthesis (Martínez-Andújar et al., 2020). Based on the following comparative transcriptome, two *NCED* genes showed higher expression in *CtDXR1* transgenic plants than wild-type plants (**Figure 6B**, **Table S3**). Therefore, these results suggested that *CtDXS1* and *CtDXR1* genes might influence the accumulation of ABA in direct and (or) indirect way.

This altered isoprenoid accumulation would directly result in the observed protection against drought stress, (or) alternatively alter plant growth. To distinguish between these possibilities, plants were grown under normal conditions (plants without drought stress) and were exposed to drought stress, respectively. Under normal conditions, transgenic plants exhibited no significant differences in morphology, but significantly higher plant growth was observed in *CtDXR1* transgenic plants (**Figure 4A**). The increase in chlorophyll content (1.46-fold higher than that in wild-type plants) was initially thought to play a role in the large phenotype of the *CtDXR1* transgenic plants. However, *CtDXS1* transgenic plants also had a 1.34-fold increase of chlorophyll content compared to wild-type plants but exhibited a relatively dwarf phenotype under normal conditions (**Figure 4A**). It was possible that the chlorophyll levels in the transgenic plants were not sufficient to produce these phenotypes in *N. benthamiana*. It was considered that a more detailed analysis of these plants could provide additional information about chlorophyll regulation and function in plants. Therefore, it was hypothesized that the main effect on growth was attributed to the alternation of phytohormonal biosynthesis, particularly that of GA_3_ and ABA. It is well known that GA_3_ and ABA antagonistically mediate many physiological processes. Some phenotypic characteristics of *CtDXR1* transgenic plants were similar to those with increased GA_3_ content, such as accumulation of biomass, increase in plant length and expansion of leaf area. Meanwhile, characteristic phenotypes such as dwarf growth and reduced biomass were observed in *CtDXS1* transgenic plants (**Figure 4A**, **Table 2**). Since the ratio between different phytohormones is more important than the absolute content (Moreno et al., 2020; Kaur et al., 2021), it is possible that the higher GA_3_/ABA ratio contributed to the strong increase in biomass (the ratio of GA_3_/ABA in *CtDXR1* transgenic plants was approximately 1:1 compared to 1:6.6 in *CtDXS1* transgenic plants).

Under drought stress conditions, both *CtDXS1* and *CtDXR1* transgenic plants exhibited significant drought tolerance (**Figure 4**). Drought stress led to an increased production of ROS, which could severely damage photosynthetic apparatus and pigments such as chlorophyll (Guadagno et al., 2017). Since ROS can be efficiently quenched by ABA-induced antioxidant and protective systems (such as PAL, SOD and POD), the abundance of ABA may be a major factor for chlorophyll protection in the transgenic lines. In addition, *β*-carotene can also reduce ROS production directly and/or indirectly, and hence is proposed to play role in drought tolerance (Moreno et al., 2020; Yu et al., 2020). However, the role of *β*-carotene in drought tolerance might be slight in this study, since the *β*-carotene content in *CtDXS1* transgenic plants was only 1.10-fold increase compared with that in wild-type plants (**Figure S4A**). The additional results obtained in **Figure S5** provided proof that two isoprenoid-type phytohormones, GA_3_ and ABA, played substantial role in plant growth and drought tolerance. Considering that both *CtDXS1* and *CtDXR1* transgenic plants showed similar trends of increased ABA (**Figure S4D**), ABA might act alone or be of more significance than *β*-carotene under drought stress.

### 3.3 The comparative transcriptome on CtDXR1 transgenic and wild-type plants

Since overexpression of *DXS* and *DXR* genes resulted in changes in isoprenoid abundance, it is possible that both DXS and DXR are rate-limiting enzymes in the MEP pathway. However, the *CtDXS1* and *CtDXR1* transgenic plants showed opposite trend of changes in GA_3_ levels, and hence it was likely that other rate-limiting enzymes for each specific isoprenoid exist. Data from other research groups (Do et al., 2016) support the hypothesis that GA levels are increased or decreased by regulating enzyme levels involved in the later GA biosynthetic steps. Interestingly, in addition to enhanced drought tolerance, accelerated plant growth was observed in *CtDXR1* transgenic plants and resulted in higher biomass accumulation. Thus, *CtDXR1* expression in crops might provide a suitable strategy to achieve a trade-off between plant growth and drought tolerance under field conditions. However, to explore its potential in crops, a better understanding of the molecular basis of its phenotype is essential, so a comparative transcriptome on *CtDXR1* transgenic plants and wild-type plants was performed. The results revealed that several genes involved in isoprenoid biosynthesis were up-regulated, including *DXS*, *KS*, and *NCED*, with *KS* being a potential regulator of GA biosynthesis (Do et al., 2016), while *DXS* and *NCED* are key genes involved in ABA biosynthesis (Thompson et al., 2007; Arias et al., 2022), suggesting that the remodeled architecture of genes involved in isoprenoid biosynthesis might contribute to the trade-off between plant hormone content. In soybean and Chinese cabbage, mutations in *KS* resulted in reduced plant height, shorter internodes, and non-headed phenotype (Li et al., 2018; Gao et al., 2020), whereas a series of dwarf phenotypes in GA-deficient mutants reverted to wild type by heterologous overexpression of *KS* (Otsuka et al., 2004), thus supporting the conclusion that increased GA levels might largely explain the developmental phenotypes observed in *CtDXR1* transgenic plants. Previous studies have shown that *DXS* and *NCED* in tobacco could induce high accumulation of ABA (Thompson et al., 2007; Arias et al., 2022), leading to the drought-resistant phenotype observed in *CtDXR1* transgenic plants.

Interestingly, the observed changes in gene expression (**Figure 6**) may be partly a direct consequence of altered phytohormonal levels. Previously, it was reported that enhanced ABA and (or) GAs contents altered the expression of genes involved in isoprenoid biosynthesis (Lv et al., 2017; Chen et al., 2022). For example, *PSY* expression in *Arabidopsis* roots (Meier et al., 2011), *NCEDs* expression in peach (Tian et al., 2020), *DXS* expression in *S. miltiorrhiza* (Yang et al., 2012b), and *GGPPS* expression in *Osmanthus fragrans* (Liu et al., 2020) were induced after treatment with ABA, respectively. Furthermore, GAs had a feedback effect on *GA3ox* expression in watermelon (Middleton et al., 2012), but no feedback effect on *GA20ox* expression in *Gibberella fujikuroi* (Tudzynski et al., 2002) and *KS* expression in sunflower (Pugliesi et al., 2011). Furthermore, characterization of the up-regulated *GGPPSs* (*GGPPS1*, *GGPPS4* and *GGPPS5*), *NCEDs* (*NCED1* and *NCED2*), *PSY* and *DXS* promoters revealed 16, 10, nine and two ABREs, respectively (**Table S6**). Although it was expected that GA response elements might be present in the promoters of *KS*, no GA regulatory elements were revealed. Therefore, the higher ABA content in *CtDXR1* transgenic plants might be mainly responsible for the feedback effect through which carotenoid content was increased.

In addition, it was previously reported that increased ABA content altered the expression of downstream stress-responsive genes (Yang et al., 2020). In our study, enhanced expression levels of *DELLA*, *LEA*, *SOD* and *ERD* were observed in transgenic plants. ABA promotes accumulation of DELLA protein, which contributes to remodeling growth and development of *Arabidopsis* seedlings (Guo et al., 2014). LEA is a key regulator of plant water loss control, and its overexpression exhibits enhanced drought tolerance (Lim et al., 2018). SOD plays a key role in protecting plants from oxidative stress, and its expression level is induced by ABA (Yu et al., 2020). ERD is rapidly activated in response to ABA and drought stress (Kariola et al., 2006). These up-regulated genes implicated that plants have evolved multi-level mechanisms for drought tolerance. Enzymatic analyses have shown that the enhanced drought tolerance of transgenic plants through ABA accumulation appears to be associated with increased activity of defense-related enzymes such as PAL, SOD, and POD. These enzymes are usually associated with induced tolerance of plants to environmental stresses (Yu et al., 2020; Qin et al., 2022). This hypothesis is consistent with previous analyses that the application of ABA together with the pathogen *Alternaria solani* induced higher expression of PAL and POD activities and defense genes (Song et al., 2011). However, this hypothesis contradicts previous analyses, which showed limited increase in POD activity in sitiens plants of tomatoes treated with ABA pulses prior to infection (Asselbergh et al., 2008). This result may be due to the diversity of each member of the multigene family. Based on the transcriptome data and real-time PCR results presented herein, the up-regulation of mRNA levels of defense-related genes might contribute to the increase in their activities.

### 3.4 Potential molecular mechanism on trade-off between plant growth and drought tolerance

Derived from these results, a molecular model was proposed to explain the compromised phenotype observed in *CtDXR1* transgenic plants. First, overexpression of *CtDXR1* promoted the accumulation of isoprenoids, such as *β*-carotene, chlorophyll, GA_3_, and ABA. Second, in *CtDXR1* transgenic plants, isoprenoid biosynthesis was remodeled at the transcriptional level (such as enhanced levels of *DXS*, *KS*, *GA20ox*, and *NCED*, or silencing of *GGPPS*), possibly due to a feedback effect. In addition, the remodeling effect might also be due to altered levels of ABA and GA_3_. Third, under normal conditions, higher GA_3_ levels or higher GA_3_/ABA ratios promoted the growth of *CtDXR1* transgenic plants. Fourth, enhanced ABA levels and/or *β*-carotene directly or indirectly protected plants in response to drought stress. In addition, ABA induced the expression of defense-related genes (such as *DELLA*, *LEA*, *SOD* and *PAL*) (Lim et al., 2018; Yu et al., 2020), which subsequently reduced the damage caused by drought, leading to improved drought tolerance (Lim et al., 2018; Wang et al., 2020; Yu et al., 2020; Qin et al., 2022).

Although the phenotypes of *CtDXS1* and *CtDXR1* transgenic plants could be largely linked to altered phytohormonal levels, it remained difficult to understand why expression of *CtDXS1* and *CtDXR1* genes from *C. tora*, but not other *DXS* and *DXR* genes from *Arabidopsis*, *P. trichocarpa*, would result in significant isoprenoid accumulation and developmental phenotypes (Simpson et al., 2016; Wei et al., 2019). One possibility is a species-specific effect, namely that similar trends in phytohormonal accumulation may not occur even with other *DXS* and *DXR* genes. Another possible explanation may lie in the different biochemical properties of the enzymes. In *C. tora*, *CtDXS1* and *CtDXR1* operate mainly in the leaves (**Figure 2A**, **Figure 2B**), where large amounts of chlorophyll accumulate. Thus, *CtDXS1* and *CtDXR1* may be less susceptible to feedback inhibition by isoprenoid products and may have a stronger positive effect on isoprenoid biosynthesis. According to our proposed mechanism, this discordance might be related to the balance of various phytohormones, and the expression structure of key genes involved in phytohormonal biosynthesis could be evaluated in the future.

## 4 Conclusion

In this study, five *DXS* genes *CtDXS1-5* and two *DXR* genes *CtDXR1-2*, which are encoded by small multigene families, were identified for the first time from the genome of *C. tora*. Among them, *CtDXS1* and *CtDXR1* were proposed to play a potential role in plant isoprenoid biosynthesis and drought tolerance, and such hypothesis was verified by the following transgenic experiments. *CtDXS1* and *CtDXR1* transgenic plants substantially influenced the isoprenoid accumulation and exhibited enhanced drought tolerance. In respect to *CtDXS1* transgenic plants, *CtDXR1* transgenic plants exhibited trade-off effect between plant growth and drought tolerance, which expands their role in future agricultural operations. Furthermore, this paper provided a molecular mechanism for the trade-off between plant growth and drought tolerance involving the remodeled structure of GA_3_/ABA biosynthesis. Overall, our study expanded the family of plant *DXSs* and *DXRs* and enhanced the understanding of the molecular mechanisms underlying the crosstalk between various plant hormones and the trade-off between plant growth and drought tolerance.

## 5 Materials and methods

### 5.1 Identification of DXS and DXR genes in *Cassia tora*

To obtain the *DXS* and *DXR* genes, all protein sequences of *Cassia tora* were downloaded from the National Center for Biotechnology Information (NCBI; http://www.ncbi.nlm.nih.gov/) and made available as a local protein database. The complete HMM maps of the DXS and DXR protein domains (PF02670 and PF02779) were also downloaded from PFam (http://pfam.sanger.ac.uk/) to identify DXS and DXR members separately. BLASTP searches were performed using HMM profiles from the Local Protein Data Bank that had 50% identity and an E-value ≤ 0.001. In the second approach, candidate was refined by CDD classification (https://www.ncbi.nlm.nih.gov/Structure/cdd/wrpsb.cgi) sequences to remove proteins without the conserved DXS (PLN02582) and DXR (PLN02696) structural domains, respectively (Marchler-Bauer et al., 2005). The corresponding results were used as queries to search again for DXS and DXR in the *C. tora* protein database. In addition, HMMER and BLAST hits were compared and the results were parsed by an in-house PERL script. The open reading frame was generated by the ORF finder (http://www.ncbi.nlm.nih.gov/gorf/gorf.htML). Phylogenetic tree construction was performed with MEGA 6.0 program (Song et al., 2022). The motif organization of protein sequences was identified using the MEME analysis at the website (http://meme.nbcr.net/meme/meme.html) (Pan et al., 2019).

### 5.2 Plant materials, total RNA isolation and cDNA preparation

The plant materials preparation, total RNA isolation and cDNA preparation were carried out based on the method reported by Liu et al. (2015). *C. tora* was grown in a greenhouse in city of Chengdu (30°41′47.04″N, 104°3′12.6″E) at 25°C, 50% humidity, and 16 h of light/8 h of darkness. Seeds of *C. tora* were collected on the 30th day after flowering in summer (09/2020), frozen in liquid N_2_ and stored at -80°C for RNA isolation. Total RNA was isolated using RNA plant-plus reagent (Omega, USA). First-strand cDNA of *C. tora* was synthesized with oligo dT-AP primers using M-MLV reverse transcriptase (TaKaRa, Japan).

### 5.3 Expression pattern analysis of CtDXS and CtDXR genes under different treatments

To analyze the expression levels of *CtDXS* and *CtDXR* genes under different stresses, sterile seedlings (7 days after germination) cultured on Murashige and Skoog (MS) solid medium were collected at 25°C with 16 h of light/8 h of darkness. For simulated drought and ABA treatments, seedlings were placed on MS medium supplemented with mannitol (200 mM) and ABA (30 μg mL^-1^) and cultured for 0, 1, 2, 3, 6, 12, 24, and 48 h, respectively. Total RNA was extracted and cDNA synthesis was performed following the same method as Liu et al. (2015). Gene-specific primer pairs for real-time PCR of *CtDXS* (CtDXS1-qF to CtDXS5-qF, CtDXS1-qR to CtDXS5-qR in **Table S7**), *CtDXR* (CtDXR1-qF, CtDXR2-qF, CtDXR1-qR and CtDXR2-qR in **Table S7**) genes and the reference gene *Elongation factor 1a* (EF1α2-qF and EF1α2-qR in **Table S7**) were designed using Primer Premier 5.0 on the basis of full-length cDNA (Liu et al., 2015). Real-time PCR was performed using SYBR Premix Ex Taq II (Takara, Japan) and LightCycler 96 system (Roche Diagnostics, Germany) based on 2 μL cDNA template in 20 μL reaction buffer using gene-specific primer pairs under the following conditions: pre-denaturation at 95°C for 1 min, followed by 40 cycles of 10 s at 95°C, 10 s at 60°C, and 20 s at 72°C. The expression levels of *CtDXS* and *CtDXR* genes were analyzed using ΔΔCT-method in LightCycler 96 software, respectively.

### 5.4 Subcellular location of CtDXS1 and CtDXR1

Firstly, primers CtDXS-1F and CtDXS-1R containing *EcoR*I and *BamH*I site, respectively, were designed to amplify the corresponding full-length CDS region of *CtDXS1* (without the stop codon). CtDXR-1F and CtDXR-1R containing *EcoR*I and *BamH*I, respectively, were designed to amplify the corresponding cDNAs of the full-length CDS region of *CtDXR1* (without the stop codon), respectively. Secondly, the *CtDXS1* fragment was introduced into the vector carrying the *GFP* fragment. Similarly, the *CtDXR1* fragment was ligated into the vector pBI121 carrying the GFP fragment to construct the vector pBI121 *CtDXR1*: *GFP*. *Agrobacterium tumefaciens* GV3101 carrying the pBI121 *CtDXS1*: *GFP* and pBI121 *CtDXR1*: *GFP* vectors were infiltrated into the young leaves of 4-week-old *Nicotiana benthamiana* plants, respectively. Leaves of *N. benthamiana* were examined four days post infiltration (dpi) using a Fluoview FV10i confocal laser scanning microscope as described previously (Qin et al., 2022). Fluorescence signals were detected using the following excitation and emission wavelengths: GFP (488 nm/510 nm) and chloroplasts (640 nm/675 nm). Images were processed with Image J software.

### 5.5 Generation of CtDXS1 and CtDXR1-transgenic plants

To analyze the functional roles of *CtDXS1* and *CtDXR1* genes in transgenic plants, *CtDXS1* and *CtDXR1* genes were overexpressed into wild-type *N. benthamiana*, respectively. First, the ORF of *CtDXS1* was amplified by PCR using primers CtDXS-2F and CtDXS-2R containing the *BamH*I and *SnaB*I sites, respectively. The ORF of *CtDXR1* was amplified by PCR using primers CtDXR-2F and CtDXR-2R containing the *Xba*I and *Sma*I sites, respectively. Subsequently, the PCR products were cloned into the pBI121 binary vector under the control of CaMV *35S* promoter. The recombinant vectors pBI-*CtDXS1* and pBI-*CtDXR1* were transformed into *A. tumefaciens* strain GV3101 and then introduced into *N. benthamiana* by *Agrobacterium*-mediated transformation, respectively. Transgenic T1 generation plants were generated and only lines showing kanamycin (50mg/L) and rifampicin (50mg/L) resistance were selected for further studies. DNA-based molecular screening of *CtDXS1* and *CtDXR1* transgenic plants was performed using primer pair PCR (CtDXS-2F and CtDXS-2R, CtDXR-2F and CtDXR-2R), respectively. Meanwhile, the expression of *CtDXS1* and *CtDXR1* in transgenic T2 plants was analyzed by RT-PCR (CtDXS-3F and CtDXS-3R, CtDXR-3F and CtDXR-3R), respectively.

### 5.6 Determination of β-carotene, ABA, GA_3_ and chlorophyll contents

The *β*-carotene, ABA, GA_3_, and chlorophyll contents of 30-day transgenic and wild-type seedlings were determined as described (Estévez et al., 2001), respectively. Total *β*-carotene was extracted from fresh sample (500 mg) with 20 mL of cooled standard solution and incubated for 16 h at 4°C. The *β*-carotene content was analyzed by HPLC using a Hypersil C18 column (4.6 mm × 150 mm) with a column temperature of 30°C. The mobile phase consisted of solvents A (0.02% butylated hydroxytoluene in methanol) and B (tetrahydrofuran). The ratio of solvent A to B in the mobile phase was set to 9:1. The elution conditions were set to a flow rate of 1 mL min^-1^, with a detection wavelength of 448 nm. The determination of each plant was carried out with three individual samples as replicates.

To determine the total chlorophyll content, 100 mg of fresh sample was extracted with 95% ethanol. Spectrophotometric quantification was performed on a Shimadzu UV-2200 spectrophotometer. To quantify the ABA and GA_3_ content of transgenic and wild-type plants, 500 mg of fresh sample was homogenized in 80% ethanol and incubated for 16 h at 4°C with continuous shaking. The supernatant was collected after centrifugation (10,000 × *g*, 15 min) and extracted with ethyl acetate. The supernatant was then dried and dissolved in 1 mL of 80% methanol, and filtered through a membrane filter (0.45 μm, 13 mm, Millipore). Finally, a mixed solution composed of ABA and GA_3_ was used as standard for the simultaneous analysis of ABA and GA_3_ by HPLC using a Hypersil C18 column (4.6 mm × 150 mm) with a column temperature of 35°C. The mobile phase consisted of methanol: 0.1% phosphoric acid (7:13, V/V) with an elution rate of 0.8 mL min^-1^. The wavelength for detection of ABA and GA3 was set to 210 nm. The determination of each plant was carried out with three individual samples as replicates.

### 5.7 Assays of PAL, SOD and POD activities

Leaf tissue (500 mg of fresh weight) was thoroughly ground in liquid nitrogen. To prepare the PAL sample, the leaf powder was homogenized with 3 mL of 100 mM ice-cold borate buffer (pH 8.8) containing 1% (w/v) polyvinylpyrrolidone (PVPP) and 0.1 mM EDTANa_2_ (Qin et al., 2022), but SOD and POD samples were prepared by homogenizing the leaf powder with 50 mM sodium phosphate buffer (pH 7.8) containing 1% (w/v) PVPP and 0.1 mM EDTANa_2_. The homogenates were centrifuged at 12,000 rpm for 20 min and the supernatant (crude extract) was used as the source of enzymes. All steps were carried out at 4°C.

PAL activity was determined according to the method of Qin et al. (2022) with some modifications. Briefly, 0.14 mL of crude extract was incubated with 3 mL of reaction buffer (100 mM boric acid (pH 8.8), 200 μM L-phenylalanine) for 30 min at 30°C. The reaction was terminated by adding 1 mL of 6N hydrochloric acid and the absorbance was measured at 290 nm using a spectrophotometer. The specific activity was expressed in units per gram of fresh weight. The reaction mixture consisted of 0.3 mL of crude extract and 3 mL of reaction solution (2% of guaiacol, 30% of H_2_O_2_, 100 mM sodium phosphate buffer (pH 7.8)), incubated for 3 min at 30°C and stopped by the addition of 10 mL of 6N hydrochloric acid. POD activity was measured as an increase in absorbance at 470 nm. SOD activity was measured using a commercial SOD assay kit (ab65354, Abcam). Reaction reagents were prepared according to the manufacturer’s instructions. After incubation at 37°C for 20 min, absorbance was measured at 450 nm. Each test was performed in triplicate.

### 5.8 Transcriptome on the wild-type and CtDXR1 transgenic plants

20-day-old *CtDXR1* transgenic and wild-type seedlings were collected, frozen in liquid nitrogen, and subsequently stored at -80°C until use. Total RNA from independent transgenic and wild-type lines was extracted. RNA-seq was performed by Biomarker Technologies (http://www.Biomarker.com.cn/) using the Illumina HiSeq™ 4000 system (Xiang et al., 2018). Clean reads were obtained after removing low-quality reads [more than 10% of bases with Q values <30 or more than 5% of Ns (where N represents ambiguous bases in the reads) from the original reads]. The clean reads were mapped to the reference genome of *N. benthamiana* “V1.0.1” (https://solgenomics.net/) using HISAT2 (Musich et al., 2021). StringTie was used for the normalization of exon reads (Pertea et al., 2015), reported as FPKM, and the number of gene reads was calculated using htseq-count (Florea et al., 2013). The IDEG6 package was used to find DEGs (Romualdi et al., 2003). According to the Benjamini-Hochberg formula, only those genes with a fold change (FC) ratio ≥ 2 and a corrected P-value < 0.05 were considered as DEGs. GO and KEGG pathway enrichment analysis of DEGs was performed using the R package (Kanehisa et al., 2008), which is based on hypergeometric distribution.

### 5.9 Statistical analysis

All experiments had at least three biological replicates, so the results are expressed as mean ± standard error. For statistical analysis, SPSS software version 19.00 (SPSS, Chicago, IL, United States) was used. Any statistically significant differences between the two groups were determined using independent *t*-tests. *p*-values < 0.05 were considered statistically significant.

## Data availability statement

The datasets presented in this study can be found in online repositories. The names of the repository/repositories and accession number(s) can be found in the article/**Supplementary Material**.

## Author contributions

HL: Writing – original draft. CT: Data curation, Writing – original draft. HQ: Writing – original draft. RJ: Writing – review & editing. QZ: Writing – review & editing. SH: Writing – review & editing. GT: Writing – review & editing. CY: Writing – review & editing. JZ: Writing – review & editing.

## Supplementary material

Supplementary Figure 1 Motifs organization and sequence logos in DXS proteins produced by MEME analysis. The DXSs in the motif analysis included AAQ84169 (*Pueraria montana* var. lobata), AMJ39459 (*Bixa orellana*), AEM42997 (*Siraitia grosvenorii*), AAS99588 (*Elaei guineensis*) and FJ455512 (*Amomum Villosum*), respectively. **(A)** motif organization of DXS proteins. The motifs are shown as different colored boxes. **(B)** the sequence logos of important motifs in DXS proteins. The height of the letter represents the degree of conservation at each position. The residue positions in the motifs are presented as the numbers on the x-axis, while the number on the y-axis represents the content measured in bits. The conserved His93, Asp294 and H499 residues for DXS catalysis were indicated with triangles, respectively. The TPP-binding domain was marked with the horizontal line and the pyridine binding DRAG domain is indicated with the double horizontal line on the top.

Supplementary Figure 2 Motifs organization and sequence logos in DXR proteins produced by MEME analysis. The DXRs in the alignment included AFP43697 (*Rhaphiolepis bibas*), BAF98290 (*Hevea brasiliensis*), QGZ18966 (*Pinus massoniana*) and ABJ80680 (*Salvia miltiorrhiza*), respectively. The NADPH-binding domain was marked with the horizontal line and the substrate binding motifs is indicated with the double horizontal line on the bottom, respectively.

Supplementary Figure 3 Expression of *CtDXS1* and *CtDXR1* in transgenic and wild-type plants. **(A)** RT-PCR results of *CtDXS1* gene in *CtDXS1* transgenic and wild-type plants, respectively. The primers (CtDXS-3F and CtDXS-3R) were used in this experiment; **(B)** RT-PCR results of *CtDXR1* gene in *CtDXR1* transgenic and wild-type plants, respectively. The primers (CtDXR-3F and CtDXR-3R) were used in this experiment.

Supplementary Figure 4 The *β*-carotene **(A)**, chlorophyll **(B)**, gibberellins (GA_3_) **(C)** and abscisic acid (ABA) **(D)** contents in transgenic and wild-type plants. Bars represent standard deviation of six independent replicates.

Supplementary Figure 5 The wild-type *N. benthamiana* plants treated by exogenous GA_3_ and ABA, respectively. For GA_3_ treatment, 14-day-old seedlings cultivated in 1/2 MS medium containing 50 μM GA_3_ were used as GA_3_ experiment group **(A)**, while those cultivated in 1/2 MS medium not containing GA_3_ were used as GA_3_ control group **(B)**. For ABA treatment, 14-day-old seedlings cultivated in 1/2 MS medium containing 100 μM ABA were used as ABA experiment group **(C)**, while those cultivated in 1/2 MS medium not containing ABA were used as ABA control group **(D)**.

Supplementary Figure 6 PAL, POD and SOD activities in *CtDXS1* transgenic **(A)**, *CtDXR1* transgenic **(B)** and wild-type plants, respectively.

Supplementary Figure 7 *Cis*-acting element analysis of potential downstream genes of ABA.
