# Supplementary material for: Differential roles of Cassia tora 1-deoxy-D-xylulose-5-phosphate synthase and 1-deoxy-D-xylulose-5-phosphate reductoisomerase in trade-off between plant growth and drought tolerance
Source: Front Plant Sci. 2023 Oct 20;14:1270396. doi: 10.3389/fpls.2023.1270396 (PMC10623318; doi:10.3389/fpls.2023.1270396)
Supplement: Supplementary file 1 [file Table_1.doc]

**Supplementary information**

**Title: Differential roles of *Cassia tora DXS* and *DXR* in trade-off between plant growth and drought tolerance**

Chunyao Tian^1,#^, Huige Quan^1,#^, Ruiping Jiang^1,#^, Qiaomu Zheng^1,#^, Sipei Huang^1^,Guodong Tan^1^, Chaoyue Yan^1^, Jiayu Zhou^1^,*, Hai Liao^1^,*

^1^ School of Life Science and Engineering, Southwest Jiaotong University, Chengdu, Sichuan, 610031, China

# Authors contributed equally.

* For Correspondence

*Corresponding authors’ information

Hai Liao

School of Life Science and Engineering

Southwest Jiaotong University

Chengdu

Sichuan

China

E-mail: spinezhou@home.swjtu.edu.cn; ddliaohai@home.swjtu.edu.cn.

**Table S1. Phenotypic statistics of wild-type seedlings treated by exogenous GA3 or not under normal condition.**

| Plants | Average lateral length of leaves (mm) | Average longitudinal lengths of leaves (mm) | Number of lateral roots | fresh weight(mg) | length of taproots(mm) |
| --- | --- | --- | --- | --- | --- |
| **+**GA3 | 10.8±0.52 | 14±0.75 | 14.2±1.04 | 111.4±7.05 | 96±3.3 |
| -GA3 | 7.8±0.59** | 11.6±0.46* | 9.8±1.31* | 86.08±5.39* | 90.6±6.7 |

Note: *, ** and *** represented statistical significance of *p*<0.05, *p*<0.01 and *p*<0.001, respectively.

**Table S1. Phenotypic statistics of wild-type seedlings treated by exogenous GA3 or not under normal condition.**

**Table S2. Phenotypic statistics of wild-type seedlings treated by ABA or not under drought stress.**

| Plants | Height (mm) | Number of lateral roots | length of taproots (mm) | fresh weight (mg) | number of leaves |
| --- | --- | --- | --- | --- | --- |
| +ABA | 71±0.37 | 9.4±0.61 | 58.6±3.0 | 36.26±1.42 | 7±0 |
| -ABA | 56.8±0.33* | 8.2±0.87 | 44.4±3.8* | 26.98±2.86* | 6.8±0.18 |

Note: *, ** and *** represented statistical significance of *p*<0.05, *p*<0.01 and *p*<0.001, respectively.

**Table S2. Phenotypic statistics of wild-type seedlings treated by ABA or not under drought stress.**

**Table S3.**

| **Gene** | **Sequence Index** | **FPKM in *CtDXR1* transgenic plants** | **FPKM in wild-type plants** | **Expression** |
| --- | --- | --- | --- | --- |
| **DXS1** | Niben101Scf00246g04005 | 167.366562 | 104.43145 | unchanged |
| **DXS2** | Niben101Scf12550g00002 | 0.740804 | 0 | up |
| **DXS3** | Niben101Scf01386g02004 | 38.368286 | 28.469376 | unchanged |
| **DXS4** | Niben101Scf03108g07001 | 0 | 0 | unchanged |
| **DXS5** | Niben101Scf04473g04001 | 4.543977 | 4.378405 | unchanged |
| **DXS6** | Niben101Scf02399g01011 | 1.84753 | 2.016183 | unchanged |
| **DXS7** | Niben101Scf06579g01017 | 5.796151 | 9.116214 | unchanged |
| **DXS8** | Niben101Scf01396g03001 | 4.20201 | 5.129266 | unchanged |
| **DXR1** | Niben101Scf01764g03022 | 44.868885 | 42.721581 | unchanged |
| **DXR2** | Niben101Scf10366g00014 | 49.665401 | 51.953987 | unchanged |
| **ISPD1** | Niben101Scf00705g01003 | 9.025553 | 12.740558 | unchanged |
| **ISPD2** | Niben101Scf06862g01003 | 9.96737 | 14.638467 | unchanged |
| **CDPMEK** | Niben101Scf04558g02010 | 10.979624 | 15.082479 | unchanged |
| **ISPF1** | Niben101Scf06180g00008 | 14.761589 | 11.736103 | unchanged |
| **ISPF2** | Niben101Scf02334g01022 | 14.132274 | 16.974686 | unchanged |
| **ISPF** | Niben101Scf06180g00014 | 14.761589 | 11.736103 | unchanged |
| **HDS1** | Niben101Scf03422g02008 | 2.449464 | 2.295076 | unchanged |
| **HDS2** | Niben101Scf09264g00009 | 53.044147 | 58.167557 | unchanged |
| **HDS3** | Niben101Scf00555g00015 | 2.934452 | 3.612519 | unchanged |
| **HDS4** | Niben101Scf11366g02020 | 66.319962 | 60.607052 | unchanged |
| **HDR1** | Niben101Scf10575g00008 | 68.9505 | 67.44117 | unchanged |
| **HDR2** | Niben101Scf33689g00006 | 77.292564 | 73.946739 | unchanged |
| **IDI1** | Niben101Scf02499g03007 | 31.541582 | 45.70752 | unchanged |
| **IDI2** | Niben101Scf01514g04018 | 37.543736 | 58.357273 | unchanged |
| **IDI3** | Niben101Scf17839g02005 | 1.057293 | 1.588043 | unchanged |
| **IDI4** | Niben101Scf01514g04014 | 30.734983 | 44.059982 | unchanged |
| **IDI5** | Niben101Scf05848g05012 | 7.881072 | 23.003735 | down |
| **GGPPS1** | Niben101Scf13710g03011 | 1.77786 | 2.668588 | unchanged |
| **GGPPS2** | Niben101Scf00929g03016 | 8.298864 | 22.897284 | down |
| **GGPPS3** | Niben101Scf10847g00001 | 2.715638 | 0.78658 | up |
| **GGPPS4** | Niben101Scf10468g00010 | 0.131892 | 0.162401 | unchanged |
| **GGPPS5** | Niben101Scf02814g03006 | 11.908916 | 31.257551 | down |
| **GGPPS6** | Niben101Scf02387g03017 | 0 | 0.660889 | unchanged |
| **GGPPS7** | Niben101Scf13146g00006 | 36.566845 | 72.360512 | unchanged |
| **GGPPS8** | Niben101Scf10847g00009 | 0 | 0 | unchanged |
| **GGPPS9** | Niben101Scf13710g03012 | 0.367951 | 0.871984 | unchanged |
| **GGPPS10** | Niben101Scf02387g03005 | 9.752061 | 2.046858 | up |
| **GGPPS11** | Niben101Scf13710g03002 | 21.102966 | 18.975933 | unchanged |
| **GGPPS12** | Niben101Scf02387g03004 | 1.519278 | 0.158626 | unchanged |
| **GGPPS13** | Niben101Scf01269g12011 | 4.304219 | 1.480958 | up |
| **GGPPS14** | Niben101Scf01269g12008 | 0 | 0 | unchanged |
| **GGPPS15** | Niben101Scf03110g02006 | 6.964937 | 10.097031 | unchanged |
| **CPS1** | Niben101Scf06819g03010 | 0.525562 | 1.001007 | unchanged |
| **CPS2** | Niben101Scf05559g00001 | 0.069287 | 0.110815 | unchanged |
| **KS1** | Niben101Scf10415g02007 | 0 | 0.106062 | unchanged |
| **KS2** | Niben101Scf02461g04014 | 6.655993 | 4.539097 | unchanged |
| **KS3** | Nicotiana_benthamiana_newGene_8393 | 0.140961 | 0 | unchanged |
| **KS4** | Niben101Scf10415g02006 | 0.032043 | 0 | unchanged |
| **KS5** | Nicotiana_benthamiana_newGene_12385 | 4.937074 | 2.360767 | up |
| **KS6** | Nicotiana_benthamiana_newGene_1235 | 0.589792 | 0 | unchanged |
| **KO1** | Niben101Scf15390g01002 | 5.07067 | 3.848997 | unchanged |
| **KO2** | Niben101Scf02322g07001 | 2.252739 | 2.191792 | unchanged |
| **KAO1** | Niben101Scf38204g00001 | 0 | 0 | unchanged |
| **KAO2** | Niben101Scf00218g04009 | 0 | 0 | unchanged |
| **KAO3** | Niben101Scf00526g00006 | 0 | 0 | unchanged |
| **KAO4** | Niben101Scf02909g07004 | 2.3768 | 2.774837 | unchanged |
| **KAO5** | Niben101Scf07482g00003 | 3.831936 | 3.452823 | unchanged |
| **GA20ox1** | Niben101Scf00428g11020 | 0.026275 | 0.047937 | unchanged |
| **GA20ox2** | Niben101Scf06024g01005 | 0.362907 | 0.802912 | unchanged |
| **GA20ox3** | Niben101Scf06404g00009 | 0.039469 | 0.116414 | unchanged |
| **GA20ox4** | Niben101Scf10027g00002 | 0.131334 | 0.387219 | unchanged |
| **GA20ox5** | Niben101Scf18902g00009 | 0.054538 | 0.586839 | unchanged |
| **GA20ox6** | Niben101Scf03245g00004 | 1.459848 | 1.284071 | unchanged |
| **GA20ox7** | Niben101Scf01036g03004 | 0 | 0 | unchanged |
| **GA20ox8** | Niben101Scf10763g00010 | 0 | 0 | unchanged |
| **GA20ox9** | Niben101Scf10196g00004 | 0.178117 | 0.110537 | unchanged |
| **GA20ox10** | Niben101Scf00188g03002 | 0.273052 | 3.240232 | down |
| **GA3ox1** | Niben101Scf05158g01008 | 0 | 0 | unchanged |
| **GA3ox2** | Niben101Scf05158g01015 | 0 | 0 | unchanged |
| **GA3ox3** | Niben101Scf05158g01041 | 0 | 0 | unchanged |
| **PSY1** | Niben101Scf07253g01008 | 99.179329 | 33.411926 | up |
| **PSY2** | Niben101Scf04664g02013 | 86.889114 | 58.215885 | unchanged |
| **PSY3** | Niben101Scf04118g01004 | 0 | 0 | unchanged |
| **PSY4** | Niben101Scf02414g03010 | 0.266377 | 0.023819 | unchanged |
| **PSY5** | Niben101Scf04020g00002 | 21.476885 | 29.382263 | unchanged |
| **PSY6** | Niben101Scf08679g04027 | 0.0 | 0.293538 | unchanged |
| **PSY7** | Niben101Scf05300g00011 | 0.0 | 0.0 | unchanged |
| **PSY8** | Niben101Scf01959g00004 | 17.631256 | 22.697952 | unchanged |
| **PSY9** | Niben101Scf01253g00004 | 2.337485 | 2.419696 | unchanged |
| **PDS1** | Niben101Scf01283g02002 | 68.144516 | 49.733093 | unchanged |
| **PDS2** | Niben101Scf14708g00023 | 27.375805 | 21.993481 | unchanged |
| **PDS3** | Niben101Scf11560g00002 | 20.103683 | 21.691011 | unchanged |
| **Z-ISO1** | Niben101Scf05548g02009 | 0.336239 | 1.252582 | down |
| **Z-ISO2** | Niben101Scf12372g00005 | 10.748878 | 10.918429 | unchanged |
| **ZDS1** | Niben101Scf02804g00014 | 36.551071 | 35.230598 | unchanged |
| **ZDS2** | Niben101Ctg11118g00001 | 0.133488 | 0.0 | unchanged |
| **ZDS3** | Niben101Scf11383g02015 | 4.501623 | 3.64467 | unchanged |
| **CRTISO1** | Niben101Scf12589g00002 | 10.031291 | 9.329317 | unchanged |
| **CRTISO2** | Niben101Scf00911g02014 | 6.382899 | 6.354841 | unchanged |
| **LYCB1** | Niben101Scf00980g07018 | 28.975859 | 31.785295 | unchanged |
| **LYCB2** | Niben101Scf06266g00002 | 21.075533 | 17.007221 | unchanged |
| **LYCB3** | Niben101Scf00980g07019 | 17.610405 | 19.027411 | unchanged |
| **HYD1** | Niben101Scf02285g01028 | 0.086953 | 1.011474 | down |
| **HYD2** | Niben101Scf01232g03010 | 68.459953 | 82.204491 | unchanged |
| **HYD3** | Niben101Scf03114g06005 | 0.049269 | 0.0 | unchanged |
| **HYD4** | Niben101Scf03114g06003 | 0.651741 | 0.316167 | unchanged |
| **ZEP1** | Niben101Scf03128g08001 | 0 | 0 | unchanged |
| **ZEP2** | Niben101Scf09317g00001 | 0.489359 | 0.629706 | unchanged |
| **ZEP3** | Niben101Scf01553g01001 | 89.033211 | 85.419434 | unchanged |
| **ZEP4** | Niben101Scf16082g00007 | 135.995682 | 91.466194 | unchanged |
| **ZEP5** | Niben101Scf03128g08003 | 0 | 0 | unchanged |
| **ZEP6** | Niben101Scf10336g03007 | 19.028793 | 41.397808 | unchanged |
| **NSY1** | Niben101Scf01750g14007 | 0.03774 | 0.596169 | down |
| **NSY2** | Niben101Scf08515g00010 | 0.480673 | 0.433471 | unchanged |
| **NCED1** | Niben101Scf02887g01010 | 0 | 0 | unchanged |
| **NCED2** | Niben101Scf00897g00001 | 0.773814 | 0.858566 | unchanged |
| **NCED3** | Niben101Scf04174g06004 | 4.986621 | 4.300068 | unchanged |
| **NCED4** | Niben101Scf10189g01008 | 0.0 | 0.0 | unchanged |
| **NCED5** | Niben101Scf10189g01002 | 0 | 0 | unchanged |
| **NCED6** | Niben101Scf02893g01002 | 0 | 0 | unchanged |
| **NCED7** | Niben101Scf02301g02007 | 0.200645 | 0.514673 | unchanged |
| **NCED8** | Niben101Scf08228g03002 | 0.0 | 0.024719 | unchanged |
| **NCED9** | Niben101Scf02887g01011 | 0 | 0 | unchanged |
| **NCED10** | Niben101Scf18822g02016 | 28.53258 | 10.981956 | up |
| **NCED11** | Niben101Scf18822g02009 | 45.46407 | 23.948835 | up |
| **CCD1** | Niben101Scf07226g05002 | 0 | 0 | unchanged |
| **CCD2** | Niben101Scf01611g07010 | 0.308798 | 0.979522 | down |
| **CCD3** | Niben101Scf18822g02009 | 45.464069 | 23.948835 | up |
| **CCD4** | Niben101Scf18822g02016 | 28.532581 | 10.981956 | up |
| **CCD5** | Niben101Scf08613g02006 | 23.102346 | 16.127584 | unchanged |
| **CCD6** | Nicotiana_benthamiana_newGene_274 | 0.691387 | 0.883362 | unchanged |
| **CCD7** | Niben101Scf01056g05003 | 0 | 0 | unchanged |
| **CCD8** | Niben101Scf00878g02006 | 0.0 | 0.157455 | unchanged |
| **CCD9** | Niben101Scf07226g05001 | 0 | 0 | unchanged |

**Table S3. The expression levels of genes involved in isoprenoid biosynthesis in *CtDXR1* transgenic and wild-type plants in the absence of drought, respectively.**

Note: DXS, 1-deoxy-D-xylulose-5-phosphate synthase; DXR, 1-deoxy-D-xylulose-5-phosphate reductoisomerase; ISPD: 2-C-methyl-D-erythritol 4-phosphate cytidylyltransferase; CDPMEK: 4-diphosphocytidyl-2-C-methyl-D-erythritol kinase; ISPF: 2-C-methyl-D-erythritol 2,4-cyclodiphosphate synthase; HDS, (E)-4-hydroxy-3-methylbut-2-enyl diphosphate synthase; HDR, 4-hydroxy-3-methylbut-2-enyl diphosphate reductase; IDI: Isopentenyl-diphosphate isomerase; GGPPS, Geranylgeranyl pyrophosphate synthase; CPS, *ent*-copalyl diphosphate synthase; KS, *ent*-kaurene synthase; KO, *ent*-kaurene oxidase; KAO, *ent*-kaurenoic acid oxidase; GA20ox, gibberellin 20 oxidase; GA3ox, gibberellin 3-oxidase; PSY, Phytoene synthase; PDS, phytoene dehydrogenase; Z-ISO, 15-*cis*-zeta-carotene isomerase; ZDS, zeta-carotene desaturase; CRTISO, carotenoid isomerase; LYCB, lycopene *beta* cyclase; HYD, *beta*-carotene hydroxylase; ZEP, zeaxanthin epoxidase; NSY, neoxanthin synthase; NCED, 9-cis-epoxycarotenoid dioxygenase; CCD, carotenoid cleavage dioxygenase.

**Table S4.**

| **Gene** | **Sequence index** | **FPKMin *CtDXR1* transgenic plants** | **FPKM in wild-type plants** | **Expression** |
| --- | --- | --- | --- | --- |
| **LEA1** | Niben101Scf03628g04006 | 2.159715 | 0 | up |
| **LEA2** | Niben101Scf08575g03004 | 3.834483 | 0 | up |
| **LEA3** | Niben101Scf03239g02003 | 5.653495 | 0.583439 | up |
| **LEA4** | Niben101Scf08020g05009 | 2.059703 | 0.193707 | up |
| **LEA5** | Niben101Scf02222g00005 | 2.398259 | 0.320616 | up |
| **LEA6** | Niben101Scf08020g06001 | 25.86163 | 4.339704 | up |
| **LEA7** | Niben101Scf07681g02008 | 21.05847 | 3.930728 | up |
| **LEA8** | Niben101Scf19584g00018 | 299.8452 | 57.08543 | up |
| **LEA9** | Niben101Scf02952g01001 | 1.374654 | 0.352378 | up |
| **LEA10** | Niben101Scf02831g07006 | 43.447 | 11.97661 | up |
| **LEA11** | Niben101Scf00862g00010 | 4.908285 | 1.365115 | up |
| **LEA12** | Niben101Scf00383g04025 | 1.167862 | 0.353718 | up |
| **LEA13** | Niben101Scf03239g04002 | 32.09366 | 10.44188 | up |
| **LEA14** | Niben101Scf08926g01019 | 1.010253 | 0.319312 | up |
| **LEA15** | Niben101Scf05764g00013 | 0.954518 | 0.322268 | up |
| **LEA16** | Niben101Scf04231g02014 | 169.3082 | 59.12655 | up |
| **LEA17** | Niben101Scf02078g05005 | 25.58217 | 11.23101 | up |
| **LEA18** | Niben101Scf01063g10013 | 100.4407 | 45.08561 | up |
| **LEA19** | Niben101Scf04717g02003 | 5.391901 | 2.503024 | up |
| **LEA20** | Niben101Scf05767g04010 | 10.11572 | 4.69328 | up |
| **LEA21** | Niben101Scf09108g00003 | 120.6033 | 61.91389 | up |
| **LEA22** | Niben101Scf00328g04016 | 6.100457 | 3.154894 | up |
| **LEA23** | Niben101Scf02030g00004 | 5.068725 | 2.910494 | up |
| **LEA24** | Niben101Scf08020g06003 | 38.91904 | 22.99141 | up |
| **LEA25** | Niben101Scf11564g01005 | 1.088918 | 2.612831 | down |
| **LEA26** | Niben101Scf01374g01004 | 9.079085 | 24.64346 | down |
| **LEA27** | Niben101Scf00646g03017 | 1.115888 | 3.081222 | down |
| **LEA28** | Niben101Scf03706g00007 | 2.25665 | 6.65811 | down |
| **LEA29** | Niben101Scf00324g02036 | 1.702452 | 6.866163 | down |
| **LEA30** | Niben101Scf00165g01004 | 2.117044 | 10.98922 | down |
| **LEA31** | Niben101Scf01802g00002 | 0.164676 | 1.803779 | down |
| **LEA32** | Niben101Scf04003g03003 | 0.155522 | 2.693257 | down |
| **DELLA1** | Niben101Scf03072g03007 | 12.37424 | 5.379349 | up |
| **DELLA2** | Niben101Scf06332g02001 | 29.05087 | 12.05806 | up |
| **DELLA3** | Niben101Scf01958g01006 | 4.105855 | 0.287167 | up |
| **DELLA4** | Niben101Scf07474g01009 | 12.70158 | 6.673162 | up |
| **DELLA5** | Niben101Scf00199g05004 | 1.522347 | 4.659738 | down |
| **DELLA6** | Niben101Scf15437g02006 | 2.821167 | 1.044641 | up |
| **DELLA7** | Niben101Scf07289g00001 | 18.834272 | 7.392887 | up |
| **DELLA8** | Niben101Scf00864g01003 | 7.645483 | 4.145772 | up |
| **DELLA9** | Niben101Scf03240g00008 | 1.976802 | 0.167081 | up |
| **DELLA10** | Niben101Scf06996g02005 | 51.83382 | 23.903742 | up |
| **DELLA11** | Niben101Scf05519g02018 | 1.246249 | 4.191426 | down |
| **DELLA12** | Niben101Scf01958g00001 | 4.209585 | 1.30939 | up |
| **DELLA13** | Niben101Scf00066g02006 | 18.286545 | 10.06411 | up |
| **DELLA14** | Niben101Scf05368g02002 | 0.164262 | 1.211257 | down |
| **DELLA15** | Niben101Scf08326g00020 | 12.914936 | 7.324481 | up |
| **DELLA16** | Niben101Scf00683g00016 | 13.219569 | 7.589237 | up |
| **SOD1** | Niben101Scf06799g01001 | 1.563515 | 3.731185 | down |
| **SOD2** | Niben101Scf03679g03009 | 205.098846 | 31.978905 | up |
| **SOD3** | Niben101Scf11604g00006 | 61.003471 | 15.017193 | up |
| **SOD4** | Niben101Scf00163g08018 | 2.937372 | 7.045545 | down |
| **SOD5** | Niben101Ctg14566g00003 | 20.44845 | 57.14846 | down |
| **SOD6** | Niben101Scf00860g03018 | 248.9898 | 89.874886 | up |
| **PAL1** | Niben101Scf04090g02004 | 3.191105 | 24.54609 | down |
| **PAL2** | Niben101Scf04090g02003 | 8.03695 | 30.50315 | down |
| **PAL3** | Niben101Scf04652g00007 | 7.416234 | 18.183905 | down |
| **PAL4** | Niben101Scf12881g00009 | 2.90096 | 26.11851 | down |
| **PAL5** | Niben101Scf12881g00010 | 10.86518 | 63.38551 | down |
| **POD1** | Nicotiana_benthamiana_newGene_5687 | 3.561622 | 0.667065 | up |
| **POD2** | Niben101Scf01676g00009 | 1.416298 | 8.269069 | down |
| **POD3** | Niben101Scf08338g00001 | 24.237717 | 78.089264 | down |
| **POD4** | Niben101Scf05682g00014 | 0.755797 | 0.30593 | up |
| **POD5** | Niben101Scf13916g00008 | 12.698192 | 2.393047 | up |
| **POD6** | Niben101Ctg12033g00001 | 3.048874 | 9.29351 | down |
| **POD7** | Niben101Scf06369g03006 | 10.972214 | 37.162312 | down |
| **POD8** | Niben101Scf01453g06001 | 4.11359 | 19.38592 | down |
| **POD9** | Niben101Scf02709g01005 | 0 | 0.832416 | down |
| **POD10** | Niben101Scf13316g00002 | 18.112228 | 6.940697 | up |
| **POD11** | Niben101Scf06439g00010 | 0.220877 | 2.159126 | down |
| **POD12** | Niben101Scf01702g01005 | 0.93055 | 2.327994 | down |
| **POD13** | Niben101Scf03861g00009 | 2.366808 | 0.258753 | up |
| **POD14** | Niben101Scf00679g05002 | 0 | 0.891589 | down |
| **POD15** | Niben101Scf01154g12010 | 0.242614 | 3.922391 | down |
| **POD16** | Niben101Scf02349g03002 | 5.686583 | 0.880731 | up |
| **POD17** | Niben101Scf01466g01036 | 1.512919 | 0.463385 | up |
| **POD18** | Niben101Scf06385g01007 | 0.646789 | 2.349624 | down |
| **POD19** | Niben101Scf09248g03013 | 0.271074 | 1.334402 | down |
| **POD20** | Niben101Scf00638g01004 | 10.75799 | 6.076228 | up |
| **POD21** | Niben101Scf01702g01012 | 0.227903 | 4.677977 | down |
| **POD22** | Niben101Scf04060g02017 | 4.695093 | 1.257007 | up |
| **POD23** | Niben101Scf03300g02001 | 0 | 10.407414 | down |
| **POD24** | Niben101Scf00087g05003 | 2.936056 | 1.127849 | up |
| **POD25** | Niben101Scf04220g02005 | 0.410075 | 1.412891 | down |
| **POD26** | Niben101Scf05227g00013 | 0 | 0.979712 | down |
| **POD27** | Niben101Scf02411g01001 | 3.081529 | 10.795843 | up |
| **POD28** | Niben101Scf02349g03003 | 3.635223 | 0.992592 | up |
| **POD29** | Niben101Scf06277g03004 | 2.548569 | 12.327415 | down |
| **POD30** | Niben101Ctg12648g00002 | 2.839642 | 0.509546 | up |
| **POD31** | Niben101Scf16024g00021 | 0 | 1.492203 | down |
| **POD32** | Niben101Scf07182g06006 | 0 | 24.09876 | down |
| **POD33** | Niben101Scf16622g01019 | 0.459141 | 12.713932 | down |
| **POD34** | Niben101Scf01721g07003 | 1.142832 | 5.919739 | down |
| **POD35** | Niben101Scf16236g00007 | 0.734598 | 2.704166 | down |
| **POD36** | Niben101Scf04626g00009 | 1.107132 | 0.209744 | up |
| **POD37** | Niben101Scf05989g01007 | 0.134964 | 0.901331 | down |
| **POD38** | Niben101Scf04551g04013 | 0.160903 | 5.689232 | down |
| **POD39** | Niben101Scf06602g00001 | 5.115388 | 21.280493 | down |
| **POD40** | Niben101Scf34323g00003 | 0.717155 | 0 | up |
| **POD41** | Niben101Scf04627g05002 | 2.274172 | 0.90128 | up |
| **POD42** | Niben101Scf06686g00001 | 1.276638 | 23.321281 | down |
| **POD43** | Niben101Scf03861g00008 | 2.071584 | 0.106448 | up |
| **POD44** | Niben101Scf06778g07006 | 8.18111 | 4.637604 | up |
| **POD45** | Niben101Scf05764g02009 | 1.06141 | 5.581743 | down |
| **POD46** | Niben101Scf08566g08014 | 13.57072 | 2.367894 | up |
| **POD47** | Niben101Scf03460g04004 | 6.722394 | 3.147749 | up |
| **ERD15** | Niben101Scf04292g03014 | 14.465115 | 5.073695 | up |

**Table S4. Differentially expressed genes (DEGs) involved in ABA signaling pathway between *CtDXR1* transgenic and wild-type plants.**

**Table S5.**

| **Gene** | **Nucleotide sequence** |
| --- | --- |
| **ERD15** | AGTCAAATGAATTCACGTACGTTTGAAACGGAGGAAGTATAATTAATGGGTTATCCCATCTTATCTGGCTAAATAAGTGTGCAGCGAAACCAAGTGCGCACAACCCACAAGCCGAAGCCATATTATATAATGCGGAACCCCACTCTTTATTATCCAACAAATTCACCATTCCCAATCTTCTTCGTCTGTTTTGTGTGTCAACAATGGCAACTTCAACCCTAAATCCTAACGCTCCAATCTTCATTCCTGCCGAATATCGTGAAGTGGAAGACTTCTCCGACCAGTGGTGGGGCCTCGTCCACTCTTCTCCCTGGTTCCGGCATTACTGGCTTCGTGAATGCTTCTCCGACTCCGACTCTACTGATGACACTACCTATGTTCCTTTTCTCCCTGATATCGAGTTTGTTTCCAACGATTTAAAGCGTAAGCACTTCGCTACTCATCAATTTACTCACACAGTCCTTTCATTTACTAATTTGAATAATTATAAGTAATTTATTTTGTTTATGTGATTAGAAGAGGAGAATAAGAACGAGTTGATTACTTTGGGGATGTTGAAATGGAACAAATCACGTGTTGCTGCTGAAATCCCAAGGTATGGGAAGAAGGCGCCGAAGATCGTGAACGTGAAAGTGAGCCCTAGGCCTATTCAGCAACCAAGGTAGGGCGAATCGCAACTCGGAAGCGCCTGACTCACTGAGTTAGCTAGATCTGTTTAGTTAATGTACATTGCTTGTTTAACTTGTTTTATATCCTGCACTTTTGTACCTTGTTTTTCCAATAAATGCCAAACCAAAAATAGATGCACAAAATGGAGCAAATGTTACTAGAACCAATATTGTAAATAGTAAATTAGATTTTAATTTTGGTTGATCTATCTAGATTTAGAATGTAAAAGTACTTCCAAAATCTCTATCAAATGAGCAATAAAATACATAAAAATCAAACTAGGAGAACCTATGGATAAGTATAATATAATAAAATGGGCAAGGTCGTCTAATAAAGAAGTGGCTTAGAGATTAGTAAGTTGATTGTTGATCCCAGTTGGTGGCTTGTTTCCCTATCTTGTGAAGAACTTTGATGACCTCTTCAGTAGTCACATTTCCAGTTACTGTCACCTTATTTAGTTGTGTATCCACATCATATGTTTCAATATCTGCCATACACCATATAAATGGAAAACAAAATCCAGATTTTCACTAATAGAATTTCAGAATACATATTCTGGTGGTAGTTGAAGAAAATTTTAGGAATTTGAAGATGTGAAAGGACTTGTCTTACCTTCCATTTTCTTGATTGCCTTCAAGATTTTCTTGATGCATTCGTCGCAGTGCAGGCCTACTTTAAGCTCCACCACCTAGTGCACAATTGAAAAGTAAATTTCAAACTGATTGATATAATAAAAAGATTGAAAGTTTGGAAAAACCTGCTATACGAATGGTTTTGAGGTACTAAGTACTAACATTGGCCATTGCAGTTGCTACGTTTCAAAACTTCTCAATATGCACAGCTGCCAAACAATCTTCCTAACTGATGCTAGTTAGAGAAGAAAATGAAGAGATGATGAATGATTTGAATGTTTTTGAAACAGTTGGAAAGATATAAAGGAAGTGGTGGGGTATAGGATTCTTTTCTTTGAG |
| **LEA8** | TGCTCTTTTTCCTTCCTCTCAGTCCTCCATGTCTATATATACTTCTCACTTCCCCTTCCAATGTATCTGCTCCCAACTGTTGTAGTTGAAGCGAAGTGTTTCAGTTCGCCGCCGTGGAAGTTTTATAAATAAATAGCTTCCGCCGCAGCGTTCCCTTGAAATTCTTTCGCTTCAAATCAAGCAAAATGGCTCGCTCTTTCTCTAACTCCAAACTCATCTCTGCTTTCGTCGTTGATACTGTTTCTTCCTTTGTTAGCAGGTAATATAACCGTGCTTTTCCAACTTTTTGTTTGGAAACGTCTAACATCTTCCCCTTTTTTTCCAAAAAGAAACTCAAATTAGTCGAGGTCGTTGAATTTTCTTTAACTATATGTTACTCTAGTCAGGTTTATTTATGTCTATACACTATATATTTCACTCCGTTCGGGTTTAACTTTGTTTTATGTTTATGTTGAGTTTGTGTAACCGGTTATAAGGGTTGGATAATAAAAGAAAGTTTAAAATATAAGTATGATGATGCAAAGAGCAAGTAGTTAACATTGAGGAAAACAGGGGAGATAAGCTCTGTGATTTCTGATTAAATTATTAATTTTGTTGATGGGACTTTCTTTCTTTCTGAGTTAAAAATGTTGACTACTAATGTTTGTGTATATGAATGAATGTTGTGCAGGCGTGGGTATGCGGCTGCATCATCAGCTAGTGTGTCAGGTGGAGTGAGAGGATCAGGGGTTAATATTATGATGAAGAAAGGTGGGGAAGAATCAAGCAAGAAGACAACATCATGGGTGCCAGACCCAGTTACTGGCTACTACAGGCCAGAGAGTCATGCTAAAGAGATTGACGCTGCTGAGCTCCGCCAGATGCTCTTGAACCACAAACCTAGACAGCACTGAATCCATGGTATGGATCAAATCATGAAATACTGTCTTGAATCTGGGGTTTTGGTTACCATACCACGTCCCATAGCGCATTGGATCTAACCATTTGATCGAACCCAACAAGAGCGAGAGAGTTTACTATGACTTACTGCGTTTATTAGAAAATATGGAGCGTCCTAGCTCGTCAATGCTTCCTGTCGTTATAGGTGAATTAATGTTGTTTTCCTGTCAGGTTTCTGTGGTGTTCTTGTTTATCTATGGGTGACCTTCTAATAAAGTTAAATTTTGCGGTATCTTTTGGTTCCTGTACTGTCTAATTTGTGGTATATTATATTTATGATTCCCCTCTCGTCCTAATTCCCTTTTCATGTCTTTCCTTATTCTCCATGCTCAACCAGGGAAATCAGTTGATAGAAGGAAAGAATAACGAAAAGAAAAAGGAAAAGATATGTGGAGATTGAATTATAAACGAATGTAAATAGTAATTTTTTGTGTGTGTTAGAGCGGGTAACTGGTAAATGCATAGAAAAAATAAAAAGAAAAAGATCAAGCATAACGTGAGCATGCGTCTAAACTTAAATGTTTGGTTACTACTATTCTAATTCTACTTATTTTATGAAAAGCAAACGGCTGGAATGTTACCGTTGCTTAAAGGTGAAGGCTGTAGGGAACAAGGAAATATCCTTTTTGTTATGTTGGTCCAATTATATGTCAACCACGAGGACTGCAACTTTGATGAAATTGGTCCTCATTTGGTGCCAGAAAGAATTAGTGAAGAATGATGAAAAGCATTTCTTCTGGAGAGAACTGCACGTCTGCACGAGCGTATTTAGAGGAGCAATAATTGAAAAGGAAGAGGAATTAAGTGGGTCATACTGGATCCGATAGTTTGGCTAACTCTAGACTTGTAAGCTTAAAACAGAAGGATCTAAACTGAGGCAAAATTCAAACGGTTGAGCATTATTTCATTTTGGCTGGTCGAACATATATATAAACAGAGAAATGGGACTGCCACTCTTTTCGCTTCCATATAGTAAGAAAATTGAGGCATCTTAAATTGAGGATGCCCTGCTAAATATATGTAGACACTGGGTTGGAAGTTATAACAAATACTCCTATGTGGGAAATGTATTTAACTTTCATATTATGTACATTGTCAATCATCTTTTACCTATTCTAGTAGTT |
| **DELLA10** | ATGGAAGCCATATTCCAAGAACAAGTTTTTCCTGGTGCAGATGCTTTCATTTTCAGGCACCCTTCTTCAATTCTAGTCGATCCAAGAGAAGATCATGTTGTACAAAATGGTACAATAGTTAATTGTCATAGTTTTGAAGATTACTCTCATGCCCATTCAAATGTTTCGGATCCTATTGTCCGAGACGGAGATTCTTCTCCTAAAGAGGAAGGGGAAGGAGAAGGAGAAGGGGACCATTCTGATGCAATGTACAAATACATAAGTCAGATGCTAATGGAAGAGGAAGATTTGGAGTATAAGCCCTGTATGTTCCACGACTGTATGGCTCTCCAAGCTGCTGAGAAATACTTCTCTGATGTCCTTCATGGATCCGATAACATTACTAATTCCCCCCAGTTTTCCGCCATTATTCCTCAGGATAAAGTTTCCTCCTCTTGTCCTGATTTCAGCAACACTTCACCTGATTCTATTGAGTCTCTTCAATCGGATCTGAATTTTGAATCCCCTGTTTCAGTGAAGAGTTTGTCTGGCTCTTTGTTGACTTCCTTTCATTCACCTAGTGGTTTGAGGGAAAAGAAAAATCATCATCGACAAGATGATGACCACCAGAGGAGTAACAAACAGTTGGCTACATTTGCTGCTGACGAATCTGAACCGTTGGAAATGTATGACAATGTGTTGTTGCTTTGTCCAAATAATCCATGTGTTCCGCCTAATGAAGTTAAAAAACCAACCAAAGTCGGAAGGCCGAGATCAGGTGGTAAGAAGCATAGCAGCAGCAAGAAGGAAATAGTAGATTTGAGAGGTCTGTTGACTCAATGTGCACAGGCAATGTCAAGCTATGATACCCGAACAGCTAATGAGTTGCTGATGCGGATAAGACAACACTCTTCGTCCCATGGGGATGGGACAGAGAGGTTGGCTCATTATCTTGCCAATGCCCTTGAAGCACGGCTGTCCAGCACAGGGACAGCGTCGTATACAGTGTTTGCATCCAGCAGGATATCAGCTGCTCACATTTTGAAAGCTTACAAGGCGTTTATCACAGCATGCCCATTCAAGTTGATGTCCAACATTTTCGCGAACAAGTATATCAAGAAGTTGATTACTGGAGGAGCACCAAGGACGATACACATAATTGATTTTGGGATTTTATATGGTTTCCAGTGGCCTTGTCTCATACAAAGTCTATCGGCCTTGAGCCGTGGGGAACCTATAAAGCTCCGCATTACTGGAGTCGAACTTCCCCAGCCTGGTTTCCGGCCAGCAGAGAGGGTTGAGGATACAGGGCGTCGTCTAAAGAAGTACTGTGACAGATTTCATGTTCCTTTCAAATTTAATGCCATAGCAAAGAAGTGGGAAAACATCACGCTTGAAGAGCTTGCAATTGACAGAAATGAGGTGCTTGTGGTTAACAGTTTGTATAGACTAGGGAACATACCTGATGAGACAGTAGTACCAACCAGTCCAAGGGATGTTGTCTTAGATTTGATCAGGAGGATCCGTCCTGATATGTTCATCCACGGAGTGGTGAACGGGACTTACAATACTCCATTCTTTCTCACACGGTTCAGGGAGGCACTTTTTCACTTCTCTACTCTGTTTGATATGTTTGAGGCTACCATGCCCCGTGAGGATGAGGACAGAAAGCTTTTCGAGGAAGAGGTTTTTGCAAGAGATGCTATGAATGTGATAGCTTGTGAAGGAACAGAGAGAGTCGAGAGACCTGAAACATACAAGCAGTGGCAACTTAGATGCGCGAGAGCTGGATTTAAACAGCTGCCACTTGACCAGGAGATTGTCAACTTTGTTAGCAACAAAGTGAGGCGGGAGTACCACAAGGACTTCTCAGTGGATGAAGATAGCCGGTGGATGCTGCAAGGATGGAAAGGACGTGTAGTTTACGCTCTCTCTTGTTGGAAGCCTGCTGAGCAGTTTTAA |
| **DELLA2** | ATGGATGCGCACCATTTCTATGATTATGGTGTGACTGGAAGCGATTTGTCATACTCTTCATATCCTACTATTAGTTCTCTACCAAATAGGCTGTCTGGAACCTCGAAATTTGATTCAGAATATTCTCCAATTTCGCCACTTGCAAACTATTTAGACTCTGAGACCTTTACCACATTAAGTGATTGCCAGGAGCAGCACAGCTCTACAGAAAATCTTTCAGGAGCTAGTTCTTCCAGTAATTCTTCGCTGGATTATAACAATTATTTCCACCGTCCAAGCCCCTCCCCGGATTGTCTTCCGGAAGGTCTGCTGGTTTTCTCTGGTGGAAATGCTGCTCTTCAGAATGTGGTTCACAGTCGGAACATGCAACATACTCTGCTGCAGTTGGAGTCTGCTTTAATGGGGCCAGACGAAGAAGTCACAAAATCTAGCCCTTCTTATGGTGGCAATAAGGCGTCACAAACATCAAATCAGAGGTCTAGGGCGTGGACCAACGAATCCAAGGTTTTGAATCAAAATGAAACTCAACGATCACAAATTTCCTTGTTCACCAAGTCAGGCAATGGAATTCTAAGTCAGGAACGGGACAAAGTACTATACAACTTGCCCTTGCATGGTGTTCCCTCTAGCAATCTGAGGCAGCTGCTTATAGCATGCGCCAGAGCTCTGGCTGAAAACAAACTAGATGATTTTGAAGTACTGGTCACCAAGGCAAGGAGTACTGTGTCCATTACTGGAGATCCAATCCAGCGTCTTGGTGCTTACATTGTAGAAGGGCTAGTAGCAAGAAAGGAGTTATCTGGAACCACCATATATAGGAGTTTGAGGTGCAAGGAGCCGGCTGGAAATGATTTGCTCTCCTACATGCATATCCTCTATGAAATATGTCCTTACCTAAAGTTTGGCTACATGGCTGCAAATGGTGCCATAGCAGAAGCATGCAGAAGCGAAAATTACGTCCACATTATTGACTTCCAAATTGCGCAAGGGACCCAGTGGATGACTCTCTTACAAGCTCTTGCTGCAAGACCTGGCGGTGCCCCCTATGTACGCATTACAGGAATTGATGATCCAGTTTCACAATATGCTCGGGGAGACGGATTGGCAGCAGTGGAGAAACGTCTATCAGCAATTTCTGAGAAATTCAACATTGCAGTAGAGTTTAATGCAGTACCAGTTTTTGCTCCAGAAGTCACTAAGGCTATGCTTGATGTAAGGCCTGGTGAGGCTGTGACAGTAAACTTCCCTTTGCAACTTCATCACACCCCTGATGAGAGTGTTGACGTGAATAACCCGAGGGATGAGCTTATTAGGATGATAAAGTCGCTTTGCCCCAAGATAGTCACTTTGGTGGAGCAAGAATCAAATACAAACACGGCCTCATTTTTCCCTAGGTTTTTAGAAGCTCTGGACTACTACCATGCAATGTTCGAGTCAATAGACGTGACCCTAGCGAGAGACAGGAAGGAGCGGATCAATGTGGAGCAGCATTGTTTGGCTAGGGATATAGTGAATGTCATAGCATGCGAGGGCAAGGAAAGGGTGGAACGTCATGAGCTACTGGGGAAGTGGAAATCCAGGTTCATGATGGCAGGTTTTCGGCAATATCCTCTGAGCTCTTATGTGAATTCAGTGATAAGGGACCTCATGAGGCGTTACTCGGAGCATTATACACTAGTGGAGAAAGATGGGGCTATGCTGTTGGGGTGGAAGGAGAGGAACCTAATCTCTGCATCTGCTTGGTACTAG |
| **SOD6** | CTTGTCTTGTCTTAATAGGGTTGGTGGAAGTGTGTACTACACAAGACTAGAGTGTACTACACAATTGAATGTGGCACCACATAATTTACTTATCGGACCACATGAAATTGCATAACTCATAATGTCGACACGTTGAAATCATTCAAAGAGCCAACATTTTACATCCCACAAAAGCAAAATCTAGAGAACTCCATACTTTGCCGATTTCTAACTTTCAGTAAACAAAATCAGAAGTCAGAAGTGATAATGATGGCCGCTGCTACTGCTTCTGCTAATTCACTGGCCTCTGCTTTTCTTCCGCGCCTAGGTCAACTTTTTGTTCCTTTTTTCTATCTTTTCTCACCCTTAAATGCAGTAGTTTGATGTTTTATTTATCAATGATGCAGGATTTCATGGGTCATCTCATCAGAGCCTACAATTGAGAACTCAAAAGGTATTTCTGGCGTATATATTTTAGATGACGTAGCCAGTAATAATTGTCTTTTCTTTGACTATTAAAGTTTACGATGAAAACAGTTTGCAAGAAAAGCTGGTTCTGGCACAATAACGGCTAAATTTGAACTCCAATCTCCTCCTTATCCCATGGTAAATTTTCAAACTTCATACACCTTTCTTTTCTTTTTTTGTTGAGTTCATATGTTCTTAATTGTCTGGATGTTGTAATAGGTCGAGCAGAATTGATATAGATAATTTATATAGCTCACGGGCCACGGCAACTATTTTGAGATTGAGGCGTTGTTATAATTGTTGTATTATGTGTTCCAATTTTTTTAACTAAACCATGTTCCCGTGTAAGCATTTGGAATTAGAAGCATAGTGACCTTAAATTTAGGGGTGGCAAATGGGCGGGTTGGGCTGAATTTGGGCGGGTCAAGATGGGTTAACTCAATAAACGAGTCATTACCCAACCTAGTCCAAAGTGTATTTGGGCTAAGATGGGCTGGTCAAGATGGGGCAAAGAATGGGTCATAACCCAACACGCCCAAGTTGATTCATGTTTTAGAATCATGCTCATCAGGCATAAAAAAGCCTTTTACCTTAAAATGTGTAAAAATATTTTGGGGGTGGGAGGGGGTTGGGGTGGATGAGTGGTGCAGAAAACAGAAAGTTGATATTATTAAAAAAATAAGTAGAAACAAAAATTTTGACGGTGGTTTGTGCGGGGTAGGGCGTAGAAAAAAAAAAGTAAGAAATATATTTCCAGTTTTAGTTTTAGTTTTAGTGTAGGGAGTGAAGAAGTTCCATTGATCATAGTTTTCCTCTAAGATGAATATCATATTTATATCACTTAATGCTTATAGATATTTAAGAAAGTCATTTCAAAGTAATGTTGTTCTCAAATCTTGGTTCATTTGATGTATTTGACTGAGACATGCATGCTAAGGCCTCTTAGGATTCATTTTTCACATTTTGGAACAGTTTTAGTTGTTGTTGTTGTTGTTGTTGTTACTCGCTAAGTATGATTTGTAGAAGGATGCAAATCTTACAATTTGTTCATAATATAAAAACTCCCCCTCCCCCTCTTTGTTTCTCCCTCTTCTGCCGGCTTCTCTGTTAGTTCGGTTTGTCTTCTTCATACACTAATATATTCGACTTCTGAATATGACATAGGATGCTTTGGAGCCTCATATGAGTAGTAGAACATTTGAGTTCCACTGGGGGAAGCATCACAGGGCTTATGTCGACAACTTAAACAAGCAAATAGATGGAACAGAACTAGATGGAAAGACACTACAAGACATAATACTTGTTACATATAATAAAGGTGCTCCCCTCCCAGCATTCAACAATGCTGCTCAGGTAAAGTTACACTTCTCCCCTTCCCGCCTTTTTTGTTTAACCACTTGAGAGGCTTCCCGAGTGGTTCATTTACTTGCTCAGTAAAGAGTTTTTCGTTATATACAATCAAGTACCTTATCAATAAATTTCTGAATATTTTTAGTATAGTCATAAAAGAAGTTTCTTTACAAGAGTCTAATATTTCATTTCTAAATGCTCCAAGTCTTTGACAGAAATTTATCATTCGATTGCTGTATTTGTTTGTAATGCACATTTTTAAGCCATCAAAGTAAGGCATTGACTATAACAACTCGGTCTGTTGGTTTATGCAATAGACAGACTTGTTATGTACAGGACTATGAAAATGGTTTATGTACTTTTGGCTATTTTTACATGTCAAGCGCCGACTTACTGAGTCCTATTATATCTTCTTTCGCATTACAGGCCTGGAATCATCAGTTTTTCTGGGAATCAATGAAGCCCAACGGAGGAGGAGAGCCATCTGGTGATTTATTGGAACTTATCAACAGAGACTTTGGTTCCTATGATGCATTTGTTAAAGAATTTAAGGCAGCTGCGGCAACACAATTTGGCTCTGGTTGGGCCTGGCTCGCGTGTGGGAAATTCTTAGCCTTTGATCTGCATGTCTATGTGTCTGTGTAACCCTTTTGTTTAAATTGACCCAAATAGCTGAAAAGTGATGCATTTGTTCCTTACTTTGTAGACAAACCTGAGGAGAAAAAGCTTGCCTTGGTGAAAACTCCTAACGCTGAAAATCCTCTTGTTTTGGGTTACACAGTGAGTATAACGTGTGCTTCTCTGTTTCTTTCGTTCATGGGGATTTTATCTTATGTACCTAGTAGTTAATATTATAGTGGAAATCTCACAACTGTGGTTTTGGTTGCAGGCGCTCCTCACCATAGACGTTTGGGAAGTAAGCCGCACACGCTCACTTGTCTTAAATCTATTTGAATTTGGGTTTTTTTTCTGACTCCCGTTGGAAGGCGGGGGGTTGAAATGATTTAGGAAGTGATGTCTTATTCTTTTACTTTTGTGTTTGACATTGCAGCATGCCTACTATCTGGACTTTCAGGTAAGCCTAGCTGATGAAACTAAAATATTTTCCTCAACACATATGTTTGAATTCTCTTAACGGTAGCACTACCATCTTTTGTTTGTAGAACCGGCGGCCTGATTACATATCCATCTTTATGGAGAAGCTCGTGTCGTGGGAAGCAGTCAGTTGTAGGCTTAAAGCAGCAACAGCTTGAGCTGCTTAGCGGGAAGACATAAAGGAGGAAGAGGCAAACATAGCAGGCACGAGAGTAAATATTTGAGGCAGAATGATTTTTGTTAAAGAGACACTATTTTCAATCCTGCTATTCTTCTCTCAGTTGAGAATTTAAATGTCTTATTATGTGCACTTTACAAGAGGGTCAAGTGATGTTCTGTATTTGGAGGACAGTATTATTTCTGTTCTTCAGCAGCTGTTAATGGCAGGAAAAAAATGAATCAATTTGAAGTGTGGGACAACAATGTAAGGACATGAATATAAATAACTCTTGCACTTTGATGCTCTAATTTTAGAATTAGAATGATATAAGACCCCCACTGAAGTCTTTCTATGATTAGAAAAAGG |
| **DELLA13** | ATGGATCCCCGGTTCAATCCGTTGCCTCATCCTGTAAACACTTTCGAGTTCGAAGATCAGATCAATTTGTCTAGCTTTGAGGAATCACTGAATCATCCTCTCAGTTATACGGATAACTATCTAGCTTTTGGCGCCCCGTACAATACATCAAGTCCAGACATTGGCAACTTTGCTCCATCCTCAAATGTGAGCTCGGAAGTGGACTCTCCAGATGATCACGACTCTGATTCGCTGTTCAAGTACCTTAACCAGATACTAATGGAGGAGAATATTGAAGATAAGCCCAGCATGTTCCATGATCCGCTTGCTCTAAAAGCTGCTGAGAAGTCCTTCTATGAAGCCCTTGGCAAGTCGTACCCTCCTTCACCCTATCGTACATCTGATTATGTTTACCATCAATTAAAAAGCCCAGACAGCATTTTCTGGAATTCCAGTGAGCATAGTACAAGCAGTAGCAATACTGGTACTAATTCCACCGATCCTCAATGGGTTGTTGATCCTGGAGAATCTAGGTTGCCTGTAGAGAGTCATCCATCCGAATACAACGTCCAGCCTTTGGTGCAGAGTAACTCAGAGAGGTCCCGTGGTTCTTTAAACAACTTCAATAATTCAAATGCCCATGTGGACTCTTTAGTAAATCCTAATGTATTTACTCATAGTGAGTCCATCTTACAGTTCAAGAGAGGGATGGAGGAAGCTAGTAAATTCCTTCCAAACCTTAGTCAATTTGTCATCGACTTGGATAAATATACATTGCCTCCAAAGATGGAGGAAGTATCCAAAGAGGCTGTCGTCAAGGTAGAGAAGGATGAGAAGAATCACTCACCTAATGGCACGAAAGGAAGAAAGCATCAGTATCCCGAGGACAGTGATCACGAGGACGAAAGGAGCAACAAACAATCAGCAATATATGCGGAGGAGGAGGTTGAGTTATCAGAGATGTTTGATAGGGTTCTGCTTTGTACAGATAAGGGGACAGGATGTGGTGATGTCAAGCGTGAGATGCCATCTGAAGTAAACAAGAGTTCAGATCAGAATGGACAAGCACATGGATCAAATGGTGGAAAAACTCGTGCTAAAAAACAAGGGACTAAAAATGAAGCTGTGGATATAAGGACTCTCTTAATTAGCTGTGCACAATCTGTTGCTGCTGACGATCGCAGGACGGCAAATGAACAGCTAAAGCATATCAGGCAGCATTCTTCACCCACAGGTGATGCATACCAGAGGCTGGCTAGCGTATTTGCAGATGGCCTTGAAGCTCGGCTGGCTGGCACTGGCACTCAACAATATGCTGCCCTTGCCCCGAAAAGGATCACAGCTGCTGAGAAATTGAAAGCATACCAGGTTTACCTTTCATCATGCCCGTTCAAGAAAATATCAATATTATTTGCGAATAAAATGATCTTCCACACAGCATCAAATGCCAGGACATTGCATCTCATAGATTTCGGTATAACATACGGTTTCCAGTGGCCAATACTCATCCAGCTTCTCTCACAGATACCTGGTGGGCCTCCAAATCTTCGCATTACTGGAATAGACCTTTCCCAACCTGGCTTCAGGCCAGCAGAAAGCTTAGAACAGACGGGGAGCCGCCTGGCAAAATATTGCGAGCGCTTTCATGTTCCATTTGAGTACAATGCGATAGCAACACAGAATTGGGAGAATATTAAACTTGAAGACTTGAAACTTGCAAGTGGCGAGACTGTTGCTGTGAACTGCCTTTTTCGTCTTAAGAACCTATTGGATGAGACAGTGGTTGTGGACAGTCCAAGGGATGCAGTTCTGAGCTTAATTAGAAAGATGAATCCAGATATCTTTGTGCAAGCTGTAGTCAATGGATCTTACAGTGCACCCTTCTTTGTCACTCGCTTCAGGGAGGCCCTCTTCCACTACTCAACTCTCTTTGACATGTTTGATGCTACTTTACCCCGTGATGATCAGCAGAGGTTGCATTTTGAACAAGAGTTTTATAGGCGCGAGGCAATGAATGTAATTGCTTGCGAAGGGTCCGAGAGGGTTGAGAGGCCTGAAACATATAAGCAATGGCAGGTCCGCTATATGAGGGCTGGATTCAAGCTGCTTCCCTTAAACCAACAACTCATGCAGAAGTTAAGGTGCAAGGTAAAGGGGGGATGCCACCGAGATTTTGTGTTTGATGAGGATGGTAATTGGATGTTACAGGGTTGGAAAGGTCGGGTAGTATGTGCTAGCTCGTGCTGGGTGCCAGCATAG |
| **KS** | CGTGCAAAAATACCTACAAATAGTTCTATCTTCCGTTCACGTTCTCCCACCACACACTTTTAGATGATGATACTTGGCTACAGAAGCAAAATCATACTACCATTTTCTCATCATAATTTGGGAAATGGAAAGTTAGGTTCATCACCTAGTAAGACACATAATTTTGATCACTGCAATAATATATTTAATAATTCAATTCTTTTGTAACTTGTGATTCTTTTTGTTGTGTGTGTGTTTTTCCTTGTTCAAAGGAAATACAATTTGCCAGAGACCATGTAAAGGAGCAAGACGCAGTTACAGTATTGCTTCATCGCTGGTAATACGAGTCCCTTTTTCTGTTCCTCTTTTTTGTGTGGGGATTTAATTGTATACTGGTTACATATTTATACACATATTATATATGAATGATACTTATATAATATATTTATTGACTATTCTTAATTTTAGTTGTTGGGTCAGCGAATATTATTAATAAAGAACTAGTTCCATCTCTGGTTTGTAGGATGATTTCGAAGAGGCAAAGGAGAGAATAAATAAAACATTTCAGAAAGTAGAATTATCTCCTTCTTCTTATGACACAGCATGGGTAGCTATGGTCCCCTCAAAAAATTCTGTAAAGCAGCCATTTTTTCCTCAGTGTTTGGATTGGATTCTTGAAAATCGAAAGAAGATGGATCTTGGGGATTAAATCCTAGCCATTCATTGCTTGTCAAGAACTCACTTTCTTCTACTTTAGCATGTTTGCTTGCTCTTCGCAAATGGGGAGTTGGAGATAACCAAGTCCAAGGAGGTAATGACATTACATTCTTCAATTAATTTCTTTTGTGAGAAAGTAATGCTGAAAAAGAAAGAAGTTTAAATATTGTATATGGATGTTTTAAAAATATTTATATAACCAGGTCATTTATTTAATTATAAAATCATTTTTAGATTTCTTGGCAACGTCTGTGTTGGGCTAAAGCCGCCCAAAAAATACTCTTAACATGATTTTATAGGTAGAAATACAAAAATAGTCCTATTCACAAGTGGTCATTCAAAAGTAGTTAGAGTTTTAAAAAACATCAAAATTTAGTCATTTTTTGTGTAAAGATAAATCTGAAAAAAAATATTGATCAGAATCCGGAAAATACTTCAATATAATATACTAGAACTCCAGTATATTATTCTGGAGTTCGGATTTTCAGTATATTTTGCCGGAACTCCAGTATATTATACTGGAGCTAGTAATGTATTCCGAACCAGCATAATATGTTGGAAGTATACACAAATGCACTGAACTCCAATATATTATGATGGAACGATATTGTTGCAGCAAAAAAATGAATATTTTCCAATGATTTGACAAACACTAACTTTTTTGAATGACCAATCCGAAAACTGGCTAGCCCATGCTATTTTTTGTCCGCACCCACATGACTTGAGTATTTATGTTGTGTATTCACTATCTCTGTCACACCTCATTTTTTACACGGATACCCCGAAGGATAAAAACGTATAAGGGAATCTTTTCAATTAAAGTGACATATTCAAAATGGAATTATTTATTTATTTAGACTCGCCGCTTGGGATATGCTTGGTTTTTAGTGTCCCAAGTCACCAGTTTATTTTGAATCCCAAATCGAAGAAATTTTTGACTGCCCAAATGGTGTCTGCAAACCAGAAATTCTAAGTAAGGAATTTTGTTGATCCGAAGGAAGGTGTTAGGTACCCTCGGATCCCGTGGTTCTAGCATGGTCGCTTAAACTATTATAATTGGCTGAAAATTCTGATTTTTGTACATGTCATCCTATTGTTCAATCTTAAATTATTTTTTACCGCTTTTAACAGTTTTAAAAGAAAACTACAATGTCGTTAAAACATGCTGCAAACCACGTTACATAAATGCACCCGCGGTTTTTGACATATTTCAACTTCATTGAGATTTGGATTTGGGTCACATAAATGTGCACCCGTACTTAGGAAGATAAAATTATTAAAGACACGCCTAAAGGGACTAGCGTATTATTATCTTTGTGGGAAACCGTGAAATTCGCTAAAAGGCATGTCTTGAATCTCTAAGTACTTTAATATGAACACTTAAGGAGGGCCACGCAATTTGTGGGTTCGGTTTGGCGCGGTGCACCTCATTTATTATTTTAAAGGGAAATTCTACAGCCAAACTATTATTTCTATTTGGATTTGTTGCTACTCGAGCCTAATGAATTAATGATACATTACTTGGGGGGTCTAAATTTATAAATCCATTCTATATTAGTTGAACCTTAGTAAATCTGTTATGCAAAACTTCCGTTTGTTCGAATATGCCTCAGAAGCTGGGCTCAATTACGAGCCCAAATCCTTATTTACCCAAAGAGTGAGCTGCTTGGGCAAACAGTAGGCCATAATTAAATAAATTCGGGTATGATAACAAGTGTGCCACTAATACACTACATTTCTGAACATTTAGATAACTATTTAACGGTGATTTTCATATTAACGTATAATACGACATGAACTGAAGCATCAAGCTGTCTTAAATTGAAAAGCAGTATTTTGAATTTTACCCTTTCATAAACACACAAAGATTAAATTCACATAGCATGTTATTATACATTTATGAGAGATACAGGACTTTCCATAAAAATGTTCATGCTACTTTGAACAACAATTCCATTCCAAATTCAAGCTTCCAACTAAGCATGATTGGAACACTTGAATCAGCTATAGTGCTGGCTAACCTTTCTACTAACTGAATGCATAAACTTATGTATCAGTTCAAATGTCCGATTACATTGATCATCAGAAATTCTAATTCATCAAAATTTAACTATTGCCACTATATAATCTAACATAAAATATACAAACAGACTGAACATGAGATTGATATGACCAATTTTACATAACAAAACACATGGACACATAGGAATTCAGAATTTGAACACTCTCTATTTTAGGCGGAAGCTTCCATTACACATTTTCTTAGCTTTGGGCCACTAGGGATTTGAAAAACACCTGGAAATGAAGCAGAATTTAAGGAAATAGGAATCAGCAATGAGAATTAGCAATCGGCTGTCGAAAATCAACATTCACACAGTAATAGGACTAATCTAGTAGCCCAGATTTATACCACACCAATATGAACCAAATCAGCCCCTTATTGACCTTAAAACAACTTGAAACCAAGCCAGGAAACCTCCAGCAATTTTCAATAACCAAACCTAAACTAAATGATGTAAATCCAAAAACCAGAATCTGAAATCAACTTAGCCTATAATGAAACAGTACTTTTTTTGAATTTTTGTTGGTTTTGAATATCTAAATGCCTTAAACTTGAAGGGAACTTTCAAATATTCAGACTAAGTAACATTTTTTATTTTTTCTTTGGATTGAAATTGTAGGAATTGAATTCCATATATGAGTTTTTGAAATGTTTTAACTAATGGACTTCATTTAGGAATTTTCAGGATTTTGTTTTCAAATATTTTAGGGAATTTTCAGAGATTTGAGATTTTTCAAAAAGCTATGAATGAAGGCACTAGGGTACCCTTTTATAGCCAAAGCCTAGGGAAACCCCTTTATTTTATTTTAACTATTGGCCCCTCTTTTAATTTTCAGTTTTTACTCGATTAGTCCCTTATTTTCTAAAAGTCTTAATATTGAAACCTTAGAATTATCCTAGTCTAGAAGAGTCTAGGGTGTTCTTGCTCACAAAGTCTTTAAGTAAACACCTAAACTGCCCTTAAAATGAATCTTGAATTACTACCACAAACCCCGGCCCCCTTTTACTCTAACAAAATTAACCATAGTTGATTGATTATAAGACTAGAAGCTTAATCAGCTATAACCAAACCACAATTAAATATGAACTACTAATATAAATAATCACGAATTCAACTCAGAGTTCTAAGACTAGTGGACACTTAACCTAACACAACTACCAGTTAATCAATCTGAATTCAAAGAAAAGGCTGAAATTAACACAATCAAAAGAAACATGCACAACTACAGCTTACATGCAATCTTATTAGTGAAATCCAATTACTAATCAAGCTACTAATAACAAAATCAAATCTGACTATATGACTGTAAGTAGAAATTAGAACCCTAAAACAGAATCCAGATTAAAAATGAAACAATAAAACTAAAAGGATACAATTAGACAAACAAGAAAAGAAAACAGAAAAAATCAATGAGTAAAAATCATGGATAAACCAATAACAACAAATAAAACCAAACAATATTTAAACAAAGCAGAAAATGCAGTCAAATAGAAATAGGGACTCACTTCTTTTTTCGGTAACCATCTTGATTGCATTCGAATTGAACCCCCAAAATAGAGTTATATGTTTATTCTTGGTAAGAACAAACCTATAACTTCATTTGGGAACTCGAATTTGGGTTTTTGACAGCTTTCTCAAAAATTGGTCGATTTTAGATTTGAAATTCAAGAAGTCTCAGAGTTTTTTGAAGGGAGTGGGGTTCAATGATTAGAAAAGAAGGCAAAGGGGAGTATAGGGTATGTTTTTGGTGGCCATTGGAGCCGGCACCGCCGTGGGCGGCGGAGCCGGCGCTCCTTTGGACAATTATTCGAGAGGTTTGGCCAGGGTTTGAGGGTTGATTTGTGAGGTGTTGGAAAGAGGAGAAGACGGGGAGTAAATGGTGTTAACTTGAACGAATTTGGAGTTTCGCGGCGGCGGTGATTTCCAGCAAGCGGCAGACAGTGGAGAAACGGTGGTGTCGTAGTGGTGTTACATCAGTGAATAGTTTGTTACGAGGAAGGTGATTATGTCTCTAGGGTTTGAGAAGGTTTTGGGACAATTTTATTATAATTGTGAGATCAAATATGGGCAGTGGGATGAGGGCTGATGGACAACTGAGATCAAATGGACGTCGAACGACGTCGTTTTGAAGTCTTCTCTGGCTGGACGGGGTATGGGGTTTGGGTTTGGGTTTGGGTTTGGGTTAAGTTTAATTGGTTCAGGGGTTTTGGTCCAGGTTTTATTGCAAGAACCGGCCCAAATCAAGGGTTTTCATCAAGAGACCCAATTTATGTATTTTCTCTTTTATATTTTTTATATATTTTTCAGATTTTGAAAATAAAAAAAAACAAACCAAAATAGATTTAACTTCCACAAAAGAAACTAATTAACCTCTAAAATAACCTAACTTGTAAATTTAAAAAAAAAAAACAAATGTAAGAATAATTATTTTTGTTATTTTTTTCTTATTTTCAATGTTAATTAATTAATAACCTATAAATATGTTAAAGTTAAGTTCTAAATGCAAACATGCTATTTTTGTATCTTTTATGAATTATATTAAGTAAAAATGCACAAAAATGCAAATAAATACAAAATCACGCAATTAAGTCCTAAAAATTCAAATAATAAAAGAAAAAAAATCCTAATTTTCAGGATTCTATAGGAGCATTTCGAGCGAGGCAAAAATCACGTGCTCACAGCTGCCCCTCTTCGTTCAAAGACATGAAGGGTTTCCGGGTAAAGATAAATGAGCAACTAAGAGATTTTTGTCTGCTTTGCATTCCATGAGATGTATTTTGAAAACTGTTTGACCGAACCCTACTTCAAAGGTTGCCTGC |
| **CCD4** | ATGGATGCCTTCTCTTCCACTTTCCTTTCTACATTATCACAACACCCTAAATCTCTTCTTTCTCCTAATTATTCTCCCAACAAATCATCTTCTCCTACTCTTAAGGCCTCCTCCGTTCGAATTGAAGAAAGGCCACAAACTACTACTAGAACAAAACAACAAGAAAAGCCAACCCCATCACCCCATAGTACTCCTCCAAAAGACACTCCCAAAAGGCAATTACCTACAAAAAAAACAGTAGGGCCATCATTTCCATCGGTTATCTTCAATGCATTTGACGATTTCGTTAACACTTTCATTGATCCTCCTTCGAAATCTTCTGTCGATCCAAAGTATATTCTTTCTAACAACTTCGCTCCGGTGGAGGAGCTTCCTCCTACTGAATGCGAGGTCGTGGCAGGCTCCCTTCCGCCTTGCCTTGACGGCGCGTACATCCGAAATGGCCCCAATCCTCAGTATCTTCCACGTGGGCCTTACCATCTTTTTGATGGAGATGGAATGCTTCATTCCATTAAAATTTCTCAAGGCAAAGCTACACTCTGTAGCCGATACGTCAAAACTTACAAATACACAGTTGAACGTGAAGCTGGTTCTCCGGTTCTCCCAAATGTATTCTCCGGTTTCAACGGTCTGACCGCCTTGGCGGCGCGTGGTGCTATCACCGCGGCTCGAGCGCTTGCAGGACAGTTCAATCCCACTAACGGCATAGGCCTAGCAAACACAAGCTTGGCTTTATTCGGAGGCAAACTTTTCGCTCTTGGTGAATCTGATTTACCGTATGCAGTAAAATTAGCCCCAGATGGTGATATTATTACCCTCGGCCGTTACGATTTTGACGGAAAGCTTTTCATGAGCATGACGGCACATCCTAAAATTGACCCAGATACTAACGAAGCTTTTGCTTTCCGTTACGGTCCAATGCCTCCTTTTTTAACTTACTTTAGAATCGAACCAAATGGTACAAAAACCCCAGACGTGCCAATATTTTCTATGACACGTCCGTCATTTCTTCATGACTTTGCAGTTACAAAGAAATATGCGATATTCTCGGACATACAAATAGTAATGAACCCACTTGAGTTCATCACCGGTGGTTCACCTGTGAGTTCCGACTCGGGGAAAATTCCCCGTCTTGGCGTGATTCCACGTTACGCCAAGGACGAGTCAGCAATGAGGTGGTTCGATGTGCCCGGGTTTAATATCGTACACGCGATTAATGCGTGGAATGAAGATGGCGGGGATACTATTGTGATGGTGGCCCCGAATATATTGTCGGTGGAGCATACGCTGGAGAGAATGGATATGATACATGCGTCTGTTGAGAAAGTGAAGATAGATTTGAAGAGTGGGATGGTGAGCAGACATCCAGTGTCGACAAGGAATCTTGATTTTGGAGTCATCAATCCTGCTTATGTTGGGAAGAAGAACAAGTACGTATAA |

**Table S5. The nucleotide sequences of eight downstream genes regulated potentially by ABA, including ERD15, LEA8, DELLA10, DELLA2, SOD6, DELLA13, KS and CCD4.**

**Table S6.**

| **Gene** | **Sequence ID** | **chromosome location** | **strand** | **The number of ABRE response elements** |
| --- | --- | --- | --- | --- |
| **NCED1** | Niben101Scf18822g02016 | Niben101Scf18822:256943,258382 | + | 1 |
| **NCED2** | Niben101Scf18822g02009 | Niben101Scf18822:259372,262941 | + | 9 |
| **DXS** | Niben101Scf12550g00002 | Niben101Scf12550:42518,46938 | + | 2 |
| **GGPPS1** | Niben101Scf10847g00001 | Niben101Scf10847:639,9317 | + | 1 |
| **GGPPS2** | Niben101Scf02814g03006 | Niben101Scf02814:288487,292527 | + | 6 |
| **GGPPS3** | Niben101Scf00929g03016 | Niben101Scf00929:359849,360949 | _ | 2 |
| **GGPPS4** | Niben101Scf02387g03005 | Niben101Scf02387:460650,461462 | + | 4 |
| **GGPPS5** | Niben101Scf01269g12011 | Niben101Scf01269:1240022,1240936 | _ | 3 |
| **PSY** | Niben101Scf07253g01008 | Niben101Scf07253:120522,124856 | + | 9 |
| **NSY** | Niben101Scf01750g14007 | Niben101Scf01750:1471651,1473150 | _ | 2 |

**Table S6. The ABREs in the promoters of the up-regulated genes involved in the isoprenoid biosynthesis.**

**Table S7.**

| **Primer** | **Sequence (5’-3’)** |
| --- | --- |
| **CtDXS-1F (*EcoR*I)** | CCGGAATTCATGGCTCTTTGCACATTCTCATTTCC |
| **CtDXS-1R (*BamH*I)** | CGCGGATCCTGACAAAACCTCTAATGCCTCTC |
| **CtDXR-1F (*EcoR*I)** | CCGGAATTCATGGCTCTGAATTTGCCTTCTCC |
| **CtDXR*-*1R (*BamH*I)** | CGCGGATCCTGCAGGAATAGGAGCCTTTTTG |
| **EF1α2-qF** | TTGAAGAACGGTGATGCAGGTA |
| **EF1α2-qR** | CACACTCTTGATGACTCCCACA |
| **CtDXS1-qF** | CGGCAGGACAAGCCTATGAA |
| **CtDXS1-qR** | TTACTCCCTTGGCAACCTCT |
| **CtDXS2-qF** | TTTCGTAAACCCAACTCACT |
| **CtDXS2-qR** | TCTTTGCTTATCATCGTCCT |
| **CtDXS3-qF** | AGTTGCGAGGGACTTGATAG |
| **CtDXS3-qR** | TGTGAGGGCTTTGCTTAGAG |
| **CtDXS4-qF** | GGCTACCCATCTGGATTTGC |
| **CtDXS4-qR** | ATCTTTGAAGCTGCGGACGA |
| **CtDXS5-qF** | ACAAATCGGTGGACCTATGC |
| **CtDXS5-qR** | TCCTCGGCCTTTCTCAGTTA |
| **CtDXR1-qF** | GCTGGCTCAAACATTAAACTTCT |
| **CtDXR1-qR** | GCTTCAGTCCTGCACAACCTACT |
| **CtDXR2-qF** | CACATGGCCTCGCCTTGATC |
| **CtDXR2-qR** | TCCTTGCCCACTCGTCGTAA |
| **CtDXS-2F (*BamH* I)** | CGGGATCCATGGCTCTTTGCACATTCTC |
| **CtDXS-2R (*SnaB* I)** | CCCTACGTATTATGACAAAACCTCTAATGCCTC |
| **CtDXR-2F (*Xba* I)** | GCTCTAGAATGGCTCTGAATTTGCC |
| **CtDXR-2R (*Sma* I)** | TCCCCCGGGTCATGCAGGAATAG |
| **CtDXS-3F** | ATGGCTCTTTGCACATTCTC |
| **CtDXS-3R** | TTATGACAAAACCTCTAATGCCTC |
| **CtDXR-3F** | ATGGCTCTGAATTTGCCTTCTC |
| **CtDXR-3R** | TCATGCAGGAATAGGAGCCTT |
| **18S-qF** | CGCTCTGGATACATTAGCATGG |
| **18S-qR** | GACAAATCGCTCCACCAACTAAG |
| **ERD15-F** | TCAACCCTAAATCCTAACGC |
| **ERD15-R** | GAGTCGGAGTCGGAGAAGCA |
| **CCD4-F** | TTCGCTCTTGGTGAATCTGA |
| **CCD4-R** | AAGGAGGCATTGGACCGTAA |
| **LEA8-F** | AAAGCAAACGGCTGGAATGT |
| **LEA8-R** | GGCACCAAATGAGGACCAAT |
| **SOD6-F** | TGGAGAAGCTCGTGTCGTGG |
| **SOD6-R** | CTCGTGCCTGCTATGTTTGC |
| **DELLA2-F** | GAGACGGATTGGCAGCAGTG |
| **DELLA2-R** | CATCCCTCGGGTTATTCACG |
| **DELLA10-F** | GCACAGGGACAGCGTCGTATA |
| **DELLA10-R** | ATGAGACAAGGCCACTGGAAA |
| **DELLA13-F** | ATCACTCACCTAATGGCACGAA |
| **DELLA13-R** | CTCACGCTTGACATCACCAC |
| **KS -F** | CGGCAGACAGTGGAGAAACG |
| **KS -R** | CAGTTGTCCATCAGCCCTCA |

**Table S7. Primers used in the paper.**
