# Supplementary figures and images for: Differential roles of Cassia tora 1-deoxy-D-xylulose-5-phosphate synthase and 1-deoxy-D-xylulose-5-phosphate reductoisomerase in trade-off between plant growth and drought tolerance
Source: Front Plant Sci. 2023 Oct 20;14:1270396. doi: 10.3389/fpls.2023.1270396 (PMC10623318; doi:10.3389/fpls.2023.1270396)

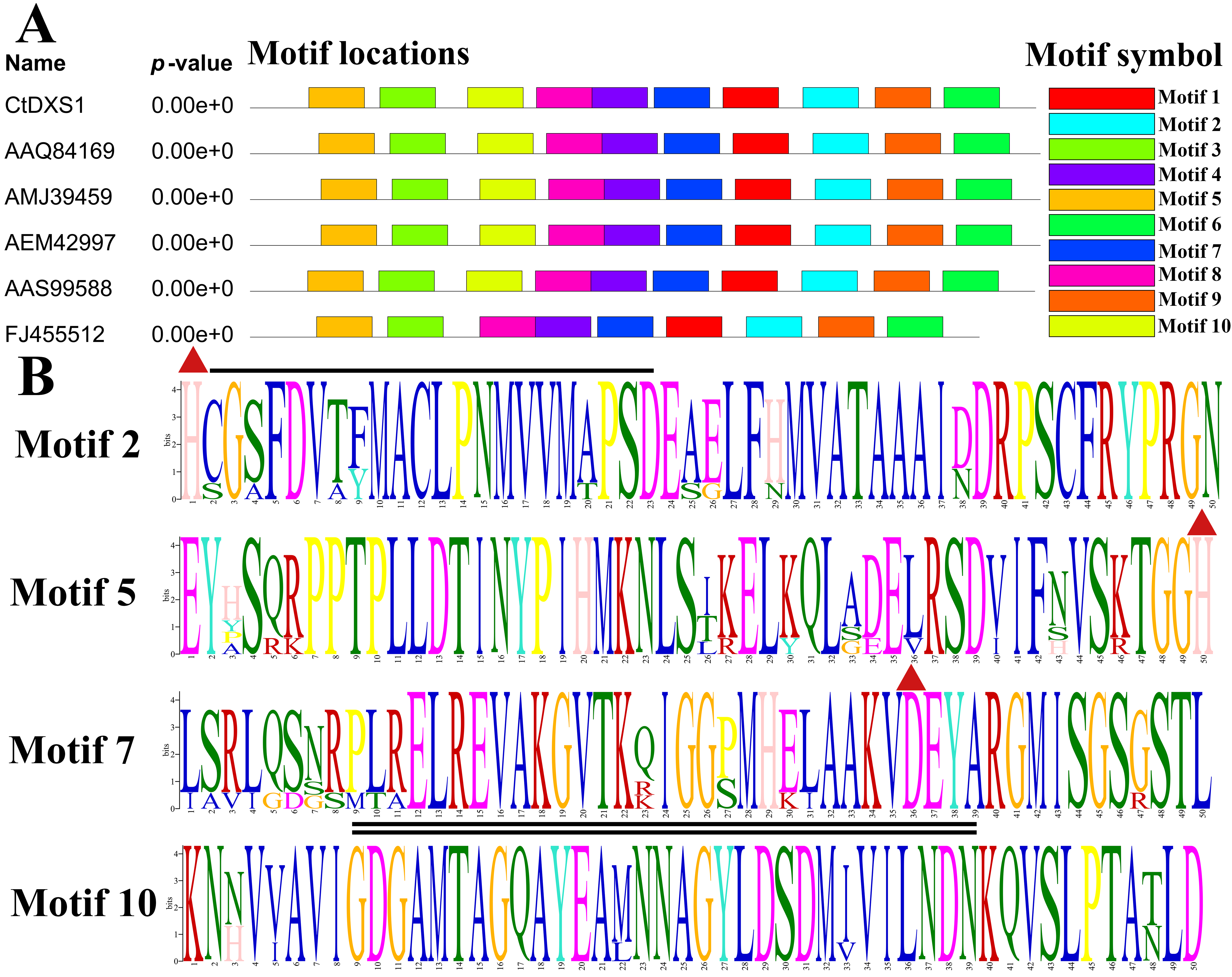

Supplement: Supplementary Figure 1 — Motifs organization and sequence logos in DXS proteins produced by MEME analysis. The DXSs in the motif analysis included AAQ84169 (Pueraria montana var. lobata), AMJ39459 (Bixa orellana), AEM42997 (Siraitia grosvenorii), AAS99588 (Elaei guineensis) and FJ455512 (Amomum Villosum), respectively. (A) motif organization of DXS proteins. The motifs are shown as different colored boxes. (B) the sequence logos of important motifs in DXS proteins. The height of the letter represents the degree of conservation at each position. The residue positions in the motifs are presented as the numbers on the x-axis, while the number on the y-axis represents the content measured in bits. The conserved His93, Asp294 and H499 residues for DXS catalysis were indicated with triangles, respectively. The TPP-binding domain was marked with the horizontal line and the pyridine binding DRAG domain is indicated with the double horizontal line on the top. [file Image_1.tif]

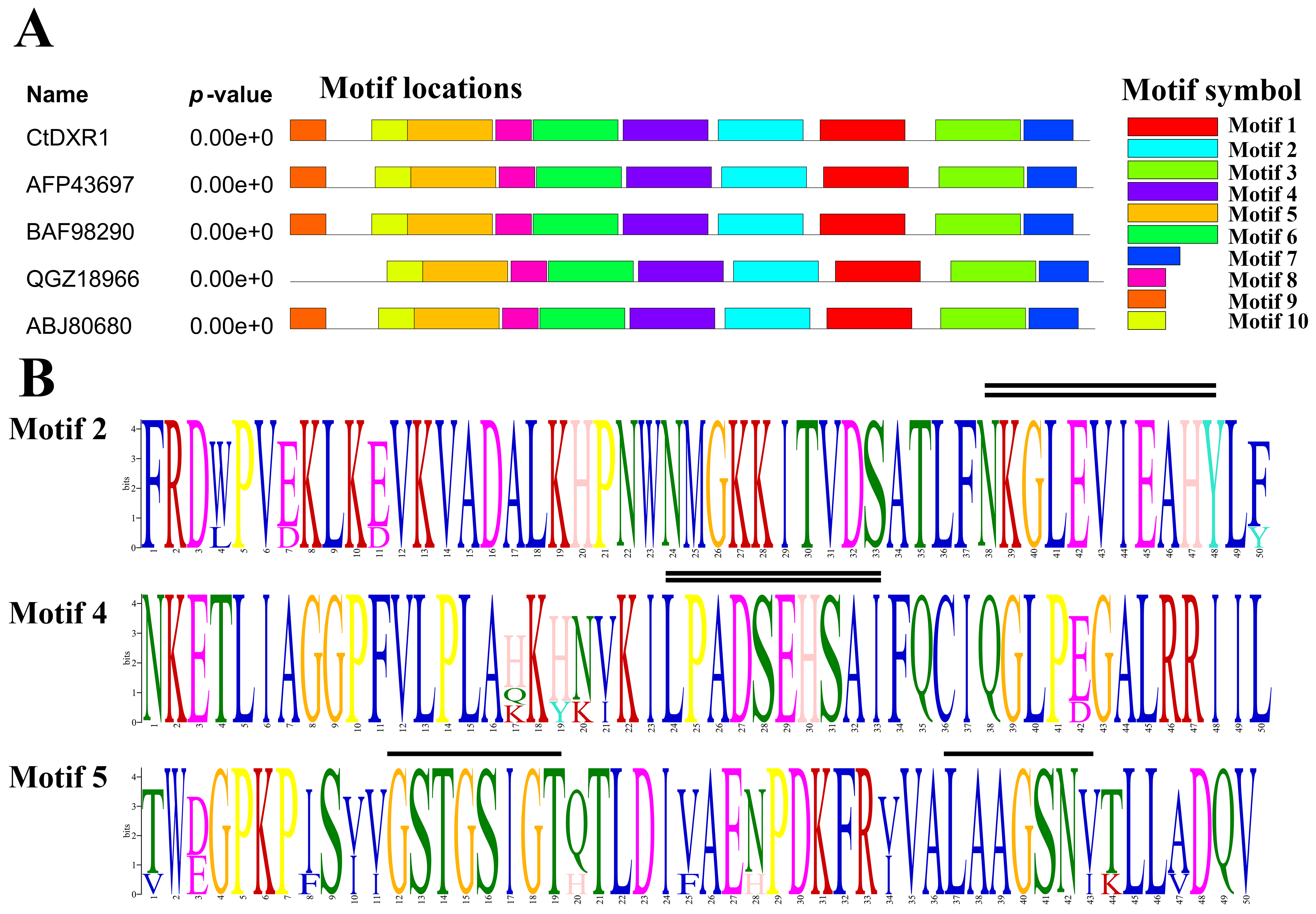

Supplement: Supplementary Figure 2 — Motifs organization and sequence logos in DXR proteins produced by MEME analysis. The DXRs in the alignment included AFP43697 (Rhaphiolepis bibas), BAF98290 (Hevea brasiliensis), QGZ18966 (Pinus massoniana) and ABJ80680 (Salvia miltiorrhiza), respectively. The NADPH-binding domain was marked with the horizontal line and the substrate binding motifs is indicated with the double horizontal line on the bottom, respectively. [file Image_2.tif]

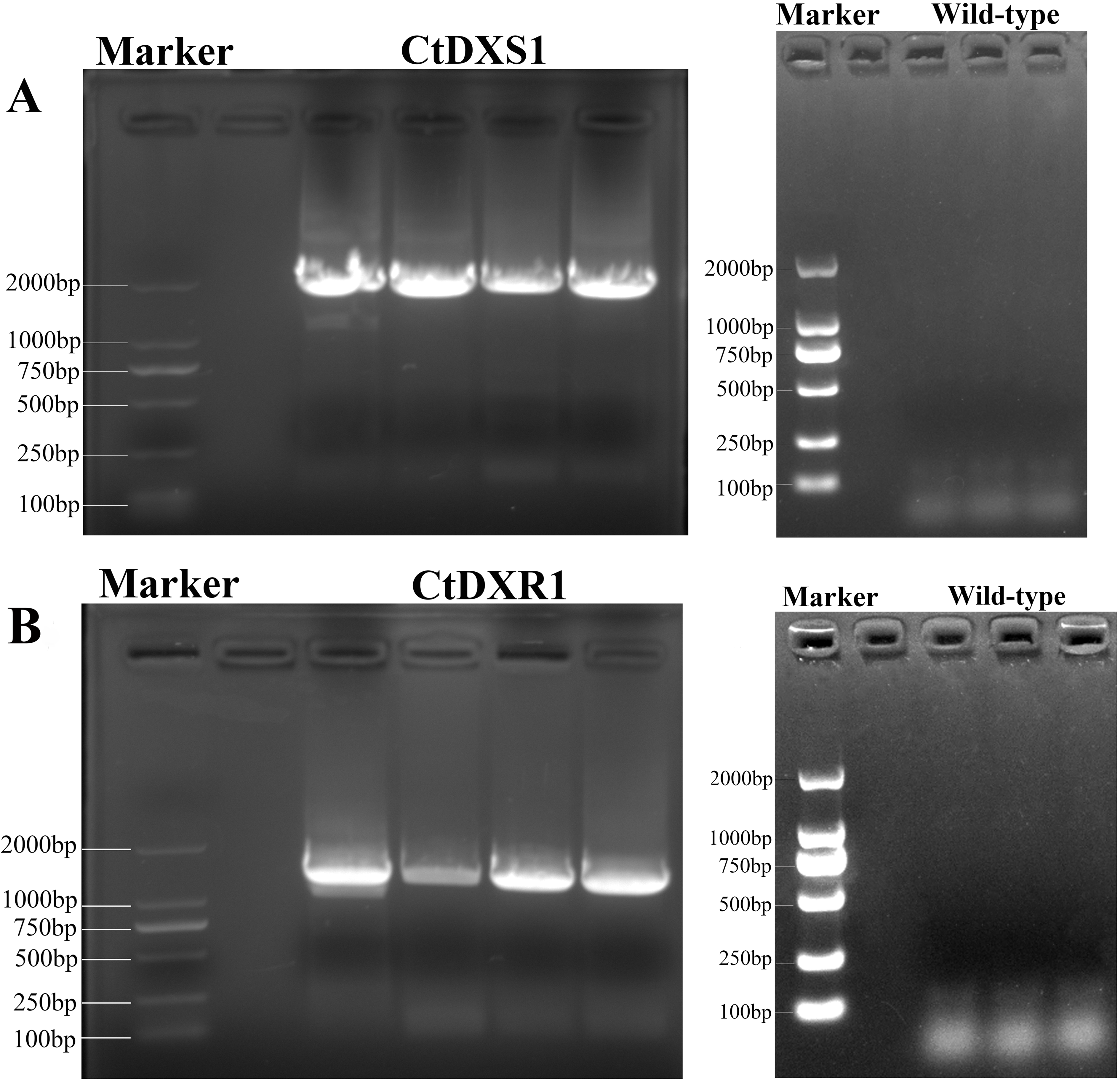

Supplement: Supplementary Figure 3 — Expression of CtDXS1 and CtDXR1 in transgenic and wild-type plants. (A) RT-PCR results of CtDXS1 gene in CtDXS1 transgenic and wild-type plants, respectively. The primers (CtDXS-3F and CtDXS-3R) were used in this experiment; (B) RT-PCR results of CtDXR1 gene in CtDXR1 transgenic and wild-type plants, respectively. The primers (CtDXR-3F and CtDXR-3R) were used in this experiment. [file Image_3.tif]

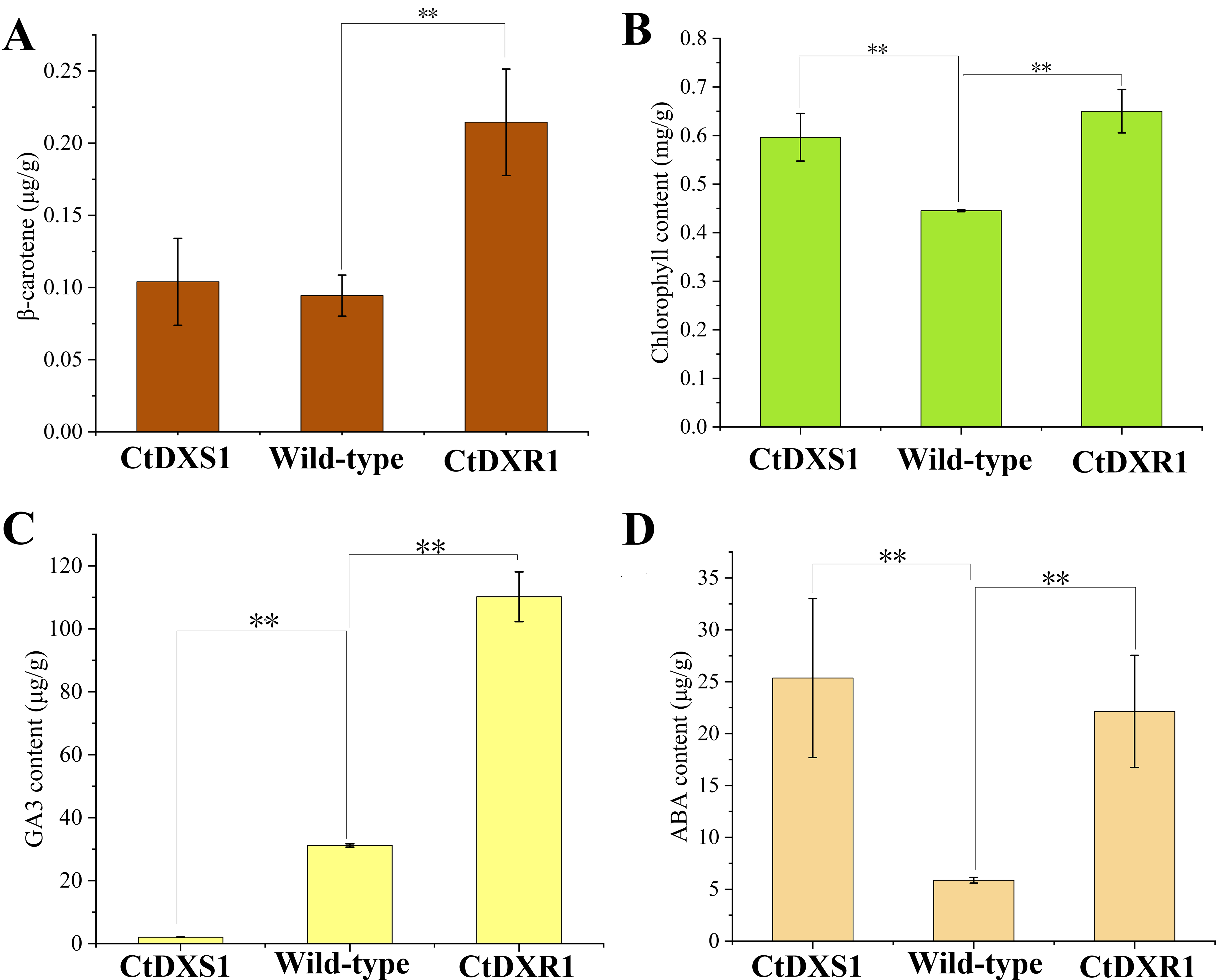

Supplement: Supplementary Figure 4 — The β-carotene (A), chlorophyll (B), gibberellins (GA3) (C) and abscisic acid (ABA) (D) contents in transgenic and wild-type plants. Bars represent standard deviation of six independent replicates. [file Image_4.tif]

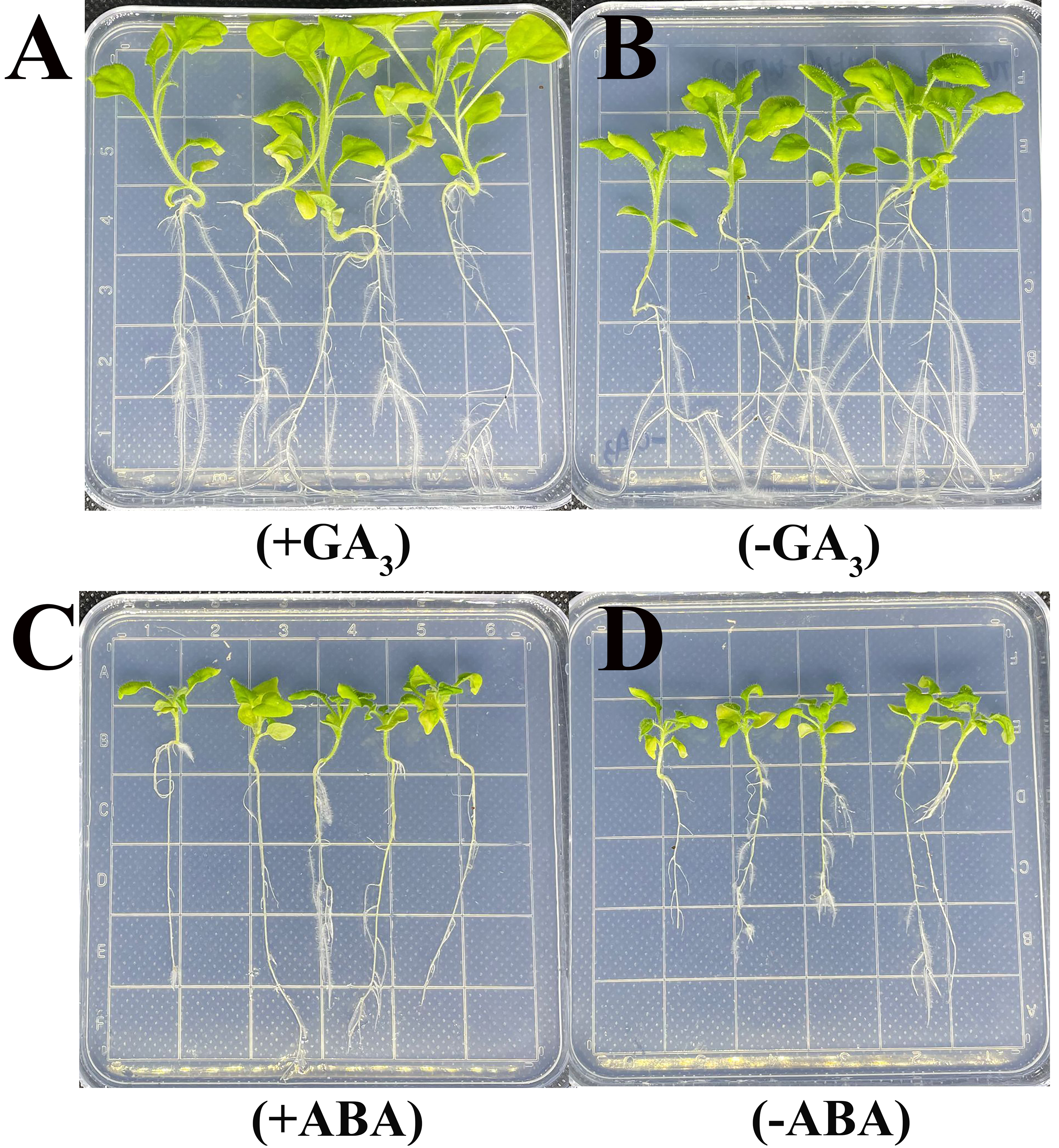

Supplement: Supplementary Figure 5 — The wild-type N. benthamiana plants treated by exogenous GA3 and ABA, respectively. For GA3 treatment, 14-day-old seedlings cultivated in 1/2 MS medium containing 50 μM GA3 were used as GA3 experiment group (A), while those cultivated in 1/2 MS medium not containing GA3 were used as GA3 control group (B). For ABA treatment, 14-day-old seedlings cultivated in 1/2 MS medium containing 100 μM ABA were used as ABA experiment group (C), while those cultivated in 1/2 MS medium not containing ABA were used as ABA control group (D). [file Image_5.tif]

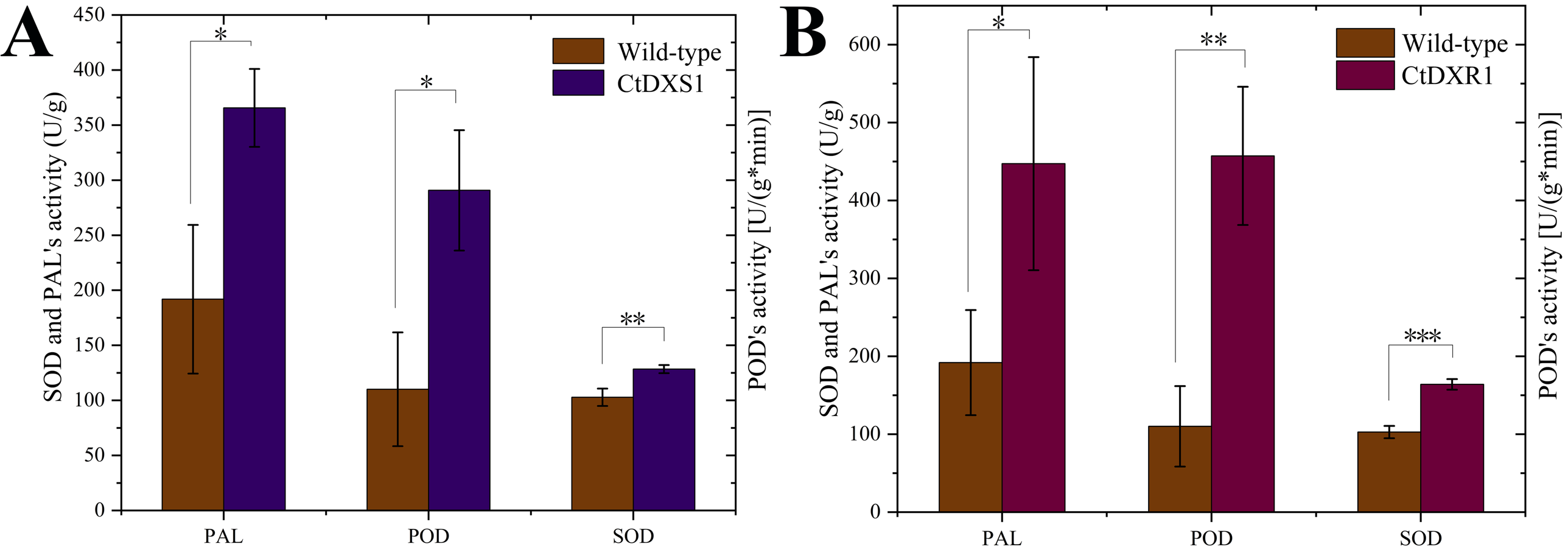

Supplement: Supplementary Figure 6 — PAL, POD and SOD activities in CtDXS1 transgenic (A), CtDXR1 transgenic (B) and wild-type plants, respectively. [file Image_6.tif]

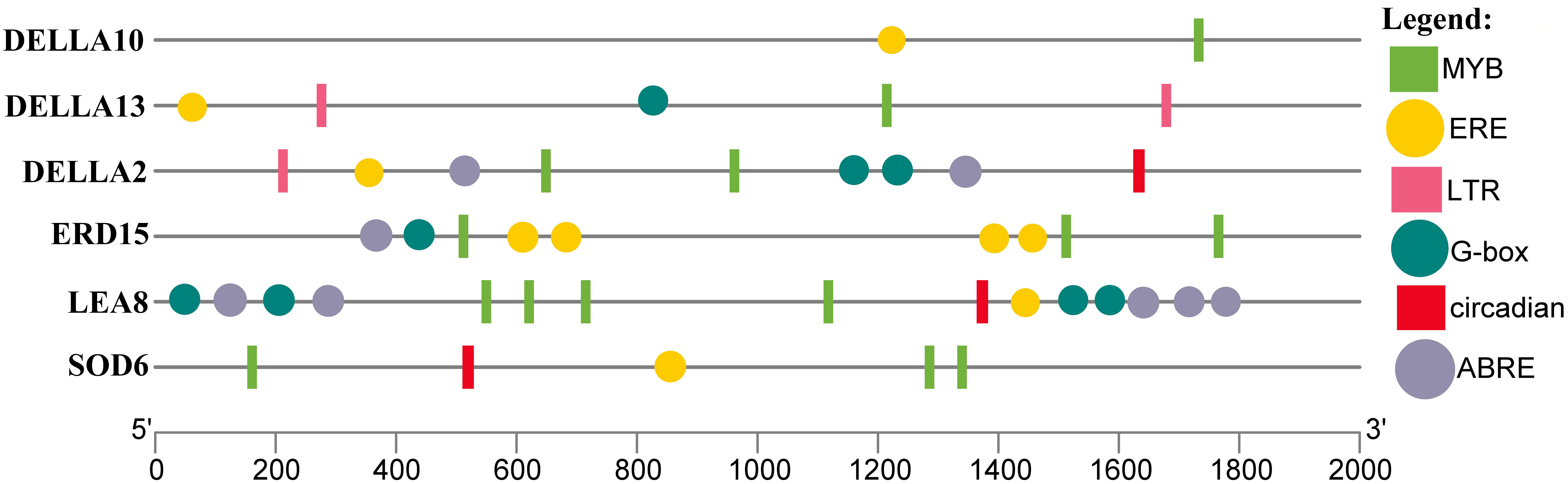

Supplement: Supplementary Figure 7 — Cis-acting element analysis of potential downstream genes of ABA. [file Image_7.tif]
